# An Amphibious Bifunctional Probe for Protein Chemical Cross-Linking

**DOI:** 10.1021/jacs.6c04886

**Published:** 2026-07-08

**Authors:** Michael Karpíšek, Lukáš Fojtík, Jan Fiala, Vojtěch Langer, Václav Matoušek, Zdeněk Kukačka, Petr Novák

**Affiliations:** † Institute of Microbiology of the Czech Academy of Sciences, Prague 14220, Czech Republic; ‡ Department of Biochemistry, Faculty of Science, Charles University, Prague 12843, Czech Republic; § CF Plus Chemicals, Brno 62100, Czech Republic

## Abstract

Chemical cross-linking mass spectrometry (CXMS) has emerged as a powerful and well-established method for probing protein structure, conformational dynamics, and protein–protein interactions, particularly in cases where classical high-resolution techniques face intrinsic limitations. The development of new cross-linking reagents with defined reactivity, activation, and compatibility with native conditions remains essential for expanding the scope and reliability of CXMS analyses. Here, we report the design and synthesis of two novel bis-hypervalent iodine reagents, called Togni cross-linking reagents TR1 and TR2, which function as efficient covalent protein cross-linkers. These reagents feature hypervalent iodine-fluoroalkyl motifs on both termini, enabling intra- and intermolecular cross-link formation. They selectively target aromatic amino acids and cysteine residues and can be activated either by ascorbate or through redox activation by thiol groups of cysteines. Moreover, these bifunctional probes can undergo transformation to form acyl fluoride, acting as a reactive acylator toward nucleophilic amino acid residues, in particular lysines. The performance of both reagents and activation modes is demonstrated on a set of structurally and functionally diverse proteins, including apomyoglobin/holomyoglobin and the small GTPase RHOA. Together, these results establish Togni cross-linking agents as versatile additions to the CXMS toolbox and highlight the potential of amphibious bis-hypervalent iodine-fluoroalkyl chemistry for studying protein structure and dynamics under mild aqueous conditions. Data is accessible via the ProteomeXchange server with the data set identifier PXD074784.

## Graphical abstract

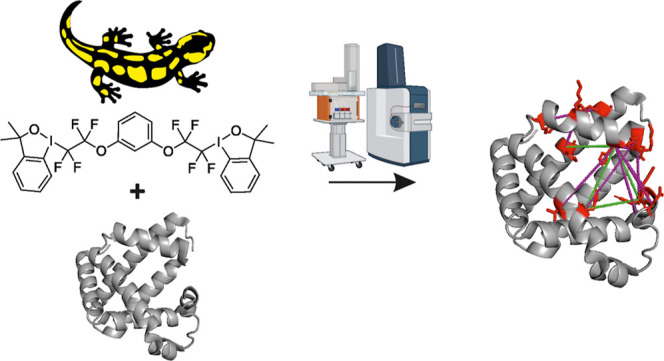

## Introduction

A common characteristic of protein cross-linking coupled with mass spectrometry (CXMS) is the identification of novel covalent bonds formed upon treatment of proteins with modifying agents. These experiments presuppose that the probability of covalent bond formation depends on the suitable proximity and reactivity of target sites within the protein landscape. Sterically protected or heavily hydrogen-bonded regions of proteins are typically modified to a lesser extent than those that are more accessible. The outcome of mass spectrometry (MS) analysis of modified proteins or protein complexes yields distance constraints between cross-linked residues, facilitating structural model design through homology modeling, molecular dynamics simulations, or AI-based approaches. Thus, the integration of chemical cross-linking and mass spectrometry significantly enhances our understanding of protein structures and dynamics.

Since the first successful attempt to obtain structural information using CXMS on a single protein two decades ago,^1^ CXMS has become an excellent complement to high-resolution structural biology techniques such as X-ray crystallography, cryo-electron microscopy (CryoEM), and nuclear magnetic resonance (NMR).^2–4^ The advantages of CXMS include low sample consumption, high sensitivity, a lack of limitations regarding protein size, the ability to study proteins in their native environments, and relatively rapid data processing. In a typical workflow, a protein or protein complex reacts with a bifunctional cross-linker, introducing a covalent bond between nearby residues.^5^ The cross-linked protein is then digested by proteases, and the resulting peptide mixture is analyzed by mass spectrometry to identify cross-linked peptides.^6^ The distance constraints obtained are subsequently utilized for structural modeling.^7–9^

Despite the fact that CXMS has proved its ability to generate distance constraints for the structural modeling of proteins and protein complexes,^10^ and a diverse repertoire of cross-linking chemistry is currently available utilizing homobifunctional^1,11,12^ heterobifunctional^13^ and photoactive^14,15^ chemical probes, the usual number of restraints obtained tends to be insufficient for reliable ab initio structural model construction if one restraint is required per ten amino acids and a closely related structural model is unavailable for machine learning. To increase the number of restraints, a higher amount of cross-linking probe can be used for the reaction. However, high concentrations of cross-linkers may induce artificial conformational changes due to disruption of local electrostatic interactions, potentially stabilizing low-populated conformational states or limiting protein flexibility. Conformational distortions can expose previously protected sites, making them accessible to the cross-linker and resulting in data artifacts.^16^ These findings underscore the need for a novel analytical workflow that provides complementary and orthogonal distance constraints while maintaining a low concentration of the cross-linker. Initially, cross-linking experiments relied on amine-reactive *N*-hydroxysuccinimide (NHS) esters, primarily targeting N-termini and lysine side chains. Although the range of available cross-linkers has expanded to include reactive probes for arginine, cysteine, aspartic acid, and glutamic acid side chains, as well as radical-based probes with broader reactivity,^11,13,15,17^ their practical application remains limited due to various challenges. Notably, there are still untapped amino acid side chains that could be targeted. The discovery of Togni reagents^18–20^ and their distinctive reactivity presents an opportunity to enhance the spatial resolution of CXMS by utilizing the side chains of cysteine, histidine, phenylalanine, tryptophan, and tyrosine for cross-linking. This notion is supported by our recent work, which demonstrated the fluoroalkylation of sulfhydryl and aromatic amino acid side chains, either with or without activation of Togni reagents by ascorbate.^21,22^ These compounds, based on a cyclic hypervalent iodine-fluoroalkyl motif, represent electrophilic reagents suitable for fluoroalkylation of biomolecules under physiological conditions.

The proposed two-step mechanism includes activation of hypervalent iodine using suitable reductants, triggering single-electron transfer (e.g., ascorbate, metal ions), followed by a subsequent attack on aromatic residues by the radical resulting from decomposition of the hypervalent iodine-fluoroalkyl reagent.^23^ Recent studies indicate modifications of all solvent-accessible sulfhydryl and aromatic amino acid side chains (Cys, Trp, His, Phe, and Tyr)^21^ in model peptides and proteins, allowing for structural model predictions of proteins and protein complexes.^24,25^ Given that the distribution of these residues in proteins is approximately 15% and they are particularly abundant in hydrophobic transmembrane proteins, cross-linking probes comprising two hypervalent iodine cores could increase the versatility of cross-linking technology and harness the potential of previously unutilized sulfhydryl and aromatic amino acid side chains. The hydrophobicity of such compounds further enhances their promise for cross-linking of membrane proteins.

To this end, we synthesized cyclic hypervalent iodine-fluoroalkyl cross-linking reagents: hydroquinone-derived Togni cross-linking reagent 1 (**TR1**) and resorcinol-derived Togni cross-linking reagent 2 (**TR2**), as shown in Figure 1. These reagents are designed to form cross-links within the side chains of cysteine, histidine, phenylalanine, tryptophan, and tyrosine. To characterize the chemical properties of Togni-based cross-linkers fully, we conducted two primary experiments using model proteins: the apo and holo forms of horse heart myoglobin (MYO) and the small human GTPase, transforming protein RHOA, were selected to define the reaction products from cross-linking reactions activated by ascorbate. Additionally, human RHOA was employed to investigate the reaction products in the absence of an external activator.

**Figure 1:**
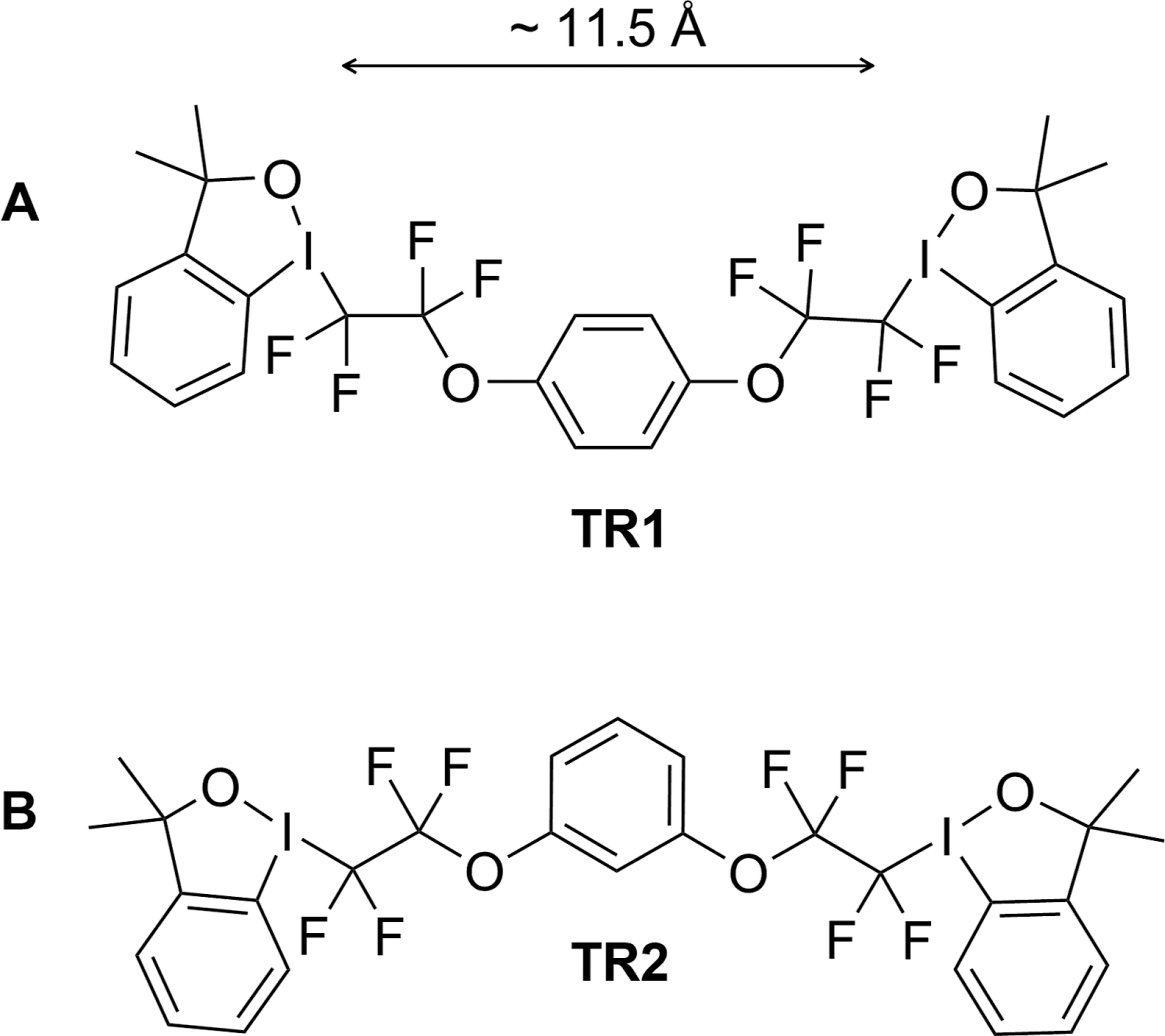
Structures of cyclic hypervalent iodine-fluoroalkyl cross-linking reagents used in this study: (A) 1,4-bis­(2-(3,3-dimethyl-1l3-benzo­[d]­[1,2]­iodaoxol-1­(3*H*)-yl)-1,1,2,2-tetrafluoroethoxy)­benzene hydroquinone alias Togni cross-linking reagent **TR1** and (B) 1,4-bis­(2-(3,3-dimethyl-1l3-benzo­[d]­[1,2]­iodaoxol-1­(3*H*)-yl)-1,1,2,2-tetrafluoroethoxy)­benzene resorcinol alias Togni cross-linking reagent **TR2**.

In the first experiment, we assessed the ability of our novel cross-linking probes, in the presence of ascorbate, to track structural rearrangements of horse MYO upon heme removal. Using Togni cross-linking reagent **TR1**, we observed two type 2 products (H64–H93 and H93–F138) and five type 0 products (F33, H36, H82, H93, and H119) on apoMYO; for holoMYO, only four type 0 products (H24, F33, F43, and H119) were detected, where type 0 indicates the addition of a cross-linker to an amino acid side chain within the protein sequence by one reactive group, with the second reactive end modified by ascorbate. For Togni cross-linking reagent **TR2**, four type 2 products (F43–H64, F43–H93, H64–H93, and H93–F138) and eight type 0 products (H24, F33, H36, F43, H36, H82, H93, H97, and H119) were observed in apoMYO tryptic peptides, while only four type 0 products (H24, F33, F43, and H119) were noted in holoMYO. Since more desired type 2 products were achieved with Togni cross-linking reagent **TR2**, it was selected for cross-linking RHOA in further experiments. However, only one type 2 product (C164–C195) and four type 0 products (C21, C112, F176, and C195) were detected. In both MYO experiments, histidine and phenylalanine were the only targets. The type 2 product for RHOA represented cysteine–cysteine cross-links, which prompted further testing of Togni cross-linking reagent **TR2** in the absence of an activator.

In the second experiment, we identified four type 2 products for RHOA (W63–C112, C112–C164, C112–C195, and C164–C195) with all but one cross-link involving cysteines. Notably, no type 0 products were observed, as ascorbate was not present. In addition to the anticipated products of the cross-linking experiments, alternative products were observed during mass spectrometry data analysis for Togni cross-linking reagents. Fragment spectra indicated that both bifunctional probes attack sulfhydryl or aromatic amino acid side chains via one reactive end, while the second fluoroalkyl radical part undergoes stepwise degradation in water to afford the corresponding acyl fluoride RCF_2_COF that forms amide bonds with neighboring lysines. The data suggest the conversion of the 2-aryloxy-1,1,2,2-tetrafluoroethyl radical to an acyl fluoride in an aqueous environment. The conversion of fluoroalkyl radicals to acyl fluoride has been reported in degradation studies on perfluoroalkylcarboxylic acids and perfluoroalkyl radicals, and intermediary unstable perfluoroalkanols are putative intermediates.^26^ Consequently, reanalysis of the data yielded additional restraints for MYO and RHOA. For Togni cross-linking reagent **TR1**, three type 1 restraints (K42–F43, H82–K87, and K96–H97) and four type 2 restraints (K42–H64, H81–K98, K45–H93, and H93–K145) were observed for apoMYO, while one type 1 restraint (K42–F43) was noted for holoMYO. For Togni cross-linking reagent **TR2**, three type 1 restraints (K42–F43, H82–K87, and H93–K96) and eight type 2 restraints (K42–H93, F43–K96, K45–H93, H64–K96, T66-H93, H82–K145, K87–F138, and H93–K98) were identified, along with one type 2 restraint (W14–K77) and two type 1 restraints (K42–F43, F43–K45) in holoMYO. Additionally, one type 1 restraint (T105-C112) was identified in the RHOA sample with ascorbic acid activation and one type 1 restraint (T105-C112) along with two type 2 restraints (G1-C112 and C164–K169) were identified in the RHOA sample without ascorbic acid activation. In addition to the products mentioned above, minor forms of the type 0 product were identified for all studied proteins as well. Finally, all distance constraints were plotted on the structural models of MYO and RHOA, confirming compliance with the maximum distance limit given the cross-linker arm length. While the original concept focused on synthesizing radical bifunctional cross-linking probes targeting aromatic amino acids, the novel probe also demonstrates reactivity toward lysine in aqueous buffers, positioning it as a highly valuable tool for the structural proteomic community. This work introduces bifunctional hypervalent iodine cross-linkers that broaden CXMS targeting to encompass sulfhydryl and aromatic residues as well as lysine, thereby facilitating an increased density of structural restraints under mild conditions.

## Experimental Section

### Materials

Unless noted otherwise, all materials were purchased from Sigma-Aldrich (USA) with the highest available purity. Mass spectrometry-grade trypsin was purchased from Promega (USA).

### Synthesis of Togni Cross-Linking Reagents

The synthesis of both Togni cross-linking reagents is based on a published synthetic strategy relying on Umpolung of an appropriate fluoroalkyl silane with a hypervalent iodine-fluoride precursor^19^ (Figure 2). In case of Togni cross-liking reagent **TR2**, resorcinol bis-trimethylsilyl ether (**1-TMS-meta**) was prepared from resorcinol **1-meta** (8.65 g, 78.6 mmol, 1 equiv, Figure S1) that was dissolved in anhydrous acetonitrile (16 mL) and mixed with hexamethyldisilazane (38 g, 235.7 mmol, 3 equiv) and a catalytic amount of saccharin (72 mg, 0.39 mmol, 0.005 equiv). The resulting mixture was refluxed for 30 min (ammonia evolution was observed) and then evaporated to dryness on a rotavap, providing clear oil of persilylated resorcinol (20 g, quantitative yield) with some traces of precipitated saccharin, which does not need to be separated. The resulting **1-TMS-meta** was used for subsequent fluoroalkylation without any other purification. In the second step, **1-TMS-meta** was fluoroalkylated to resorcinol-bis-fluoroalkyl bromide **2-meta** (Figure S2). A 2-necked 50 mL round-bottom flask equipped with a septum was charged with cesium fluoride (8.95 g, 58.95 mmol, 3 equiv) and dried with a heat gun under high vacuum. The flask was allowed to reach room temperature and was backfilled with nitrogen. Prior to starting the reaction, it was evacuated again and charged with tetrafluoroethylene gas from the attached balloon. To the flask, cooled in an ice-salt-water bath (−10 °C initial temperature), was sequentially injected anhydrous dimethylformamide (DMF, 15 mL), Halon 2402 (15.3 g, 58.95 mmol, 3 equiv), and starting material **1-TMS-meta** (5.0 g, 19.6 mmol, 1 equiv). The resulting mixture was stirred for 20 h, gradually reaching room temperature. The next day, the reaction mixture was poured into water (150 mL) and extracted with cyclohexane (4 × 40 mL). The pooled organic fractions were washed with 10% sodium hydroxide, water, and brine, dried over anhydrous magnesium sulfate, filtered, and concentrated to dryness, affording the bis-fluoroalkyl bromide **2-meta** product as a colorless oil (6.91 g, 75% yield). In the third step, resorcinol-derived bis-fluoroalkyl bromide **2-meta** was converted to the corresponding bis-fluoroalkyl silane **3**-**meta** (Figure S3).

**Figure 2:**
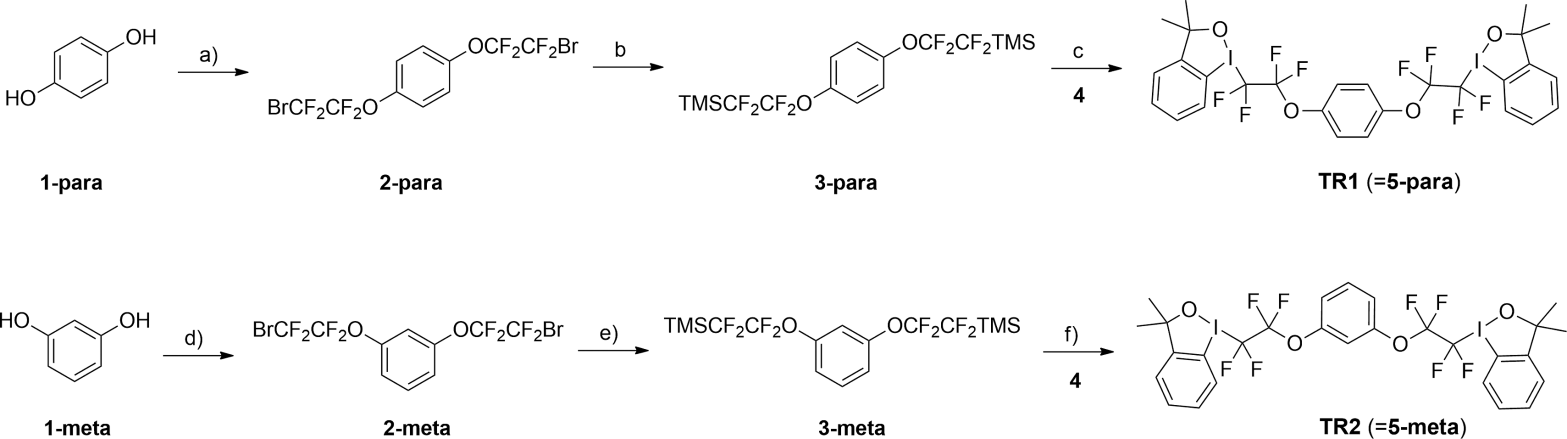
Scheme of synthesis of Togni cross-linking reagents (**TR1** and **TR2**). (a) 2.05 equiv of NaH, DMSO, r.t., 20 min, then 2 equiv of Halon 2402, 15 °C to r.t., overnight, 23%; (b) 3 equiv of TMSCl, 2.1 equiv of *i*-PrMgCl*LiCl, −78 °C to r.t., THF, 3 h, 85%; (c) 3 equiv of 1-fluoro-3,3-dimethylbenziodaoxole 4, 3 mol % TBAT, MeCN, −35 °C to r.t., 3 h, 37%; (d) 5 equiv of HMDS, 0.5 mol % saccharin, MeCN, reflux, 30 min, evaporation, quant.; then 3 equiv of CsF, DMF, 3 equiv of Halon 2402, overnight, r.t., 75%; (e) 3 equiv of TMSCl, 2.6 equiv of *i*-PrMgCl*LiCl, −78 °C to r.t., THF, 3 h, 85%; (f) 3 equiv of 1-fluoro-3,3-dimethylbenziodaoxole, 3 mol % TBAT, MeCN, −35 °C to r.t., 3 h, 38%.

The resorcinol-derived bis-fluoroalkyl bromide **2-meta** (2.1 g, 4.41 mmol, 1 equiv) was dissolved in anhydrous tetrahydrofuran (7 mL), and trimethylsilyl chloride was added (1.44 g, 11.47 mmol, 3 equiv). To the resulting solution, which was prechilled to −78 °C, was dropwise added a solution of the isopropylmagnesium chloride-lithium chloride complex (0.6 M in THF, 19.2 mL, 2.6 equiv), the so-called TurboGrignard reagent. The resulting turbid solution was stirred at −78 °C for 1 h and then gradually allowed to reach room temperature over the course of 3 h. The reaction mixture was concentrated to dryness on a rotavap and then suspended in a mixture of diethyl ether and pentane (1/1 v/v, 80 mL), and the resulting slurry was slowly added to 150 mL of an ice-cold, well-stirred solution of phosphate buffer at pH = 3. The organic phase was separated, and the aqueous phase was extracted with a mixture of pentane/diethyl ether (1/1 v/v, 3 × 60 mL). The pooled organic fractions were washed with brine, dried over anhydrous magnesium sulfate, filtered, and concentrated to dryness, affording the bis-fluoroalkyl silane product **3-meta** as a slightly yellowish oil (1.73 g, 85% yield). The product was taken into the next step without any additional purification. The last reaction of the synthesis was the Umpolung of bis-fluoroalkylsilane **3-meta** with hypervalent iodine-fluoride precursor **4**, leading to Togni cross-linking reagent **TR2** (Figure S4). A hypervalent iodine-fluoride precursor (“alcohol type” fluoroiodane **4**, 840 mg, 3 mmol, 3 equiv) was charged into a Schlenk flask under Ar, suspended in anhydrous acetonitrile (5 mL), and a tetrabutylammonium difluoridotriphenylsilicate catalyst (16.2 mg, 0.03 mmol, 0.03 equiv) was added at once. The resulting mixture was chilled to −35 °C, leading to partial reprecipitation of fluoroiodane **4**. To the resulting mixture was slowly added (over 1 h) a solution of **3-meta** (454 mg, 1 mmol, 1 equiv) in anhydrous acetonitrile (1 mL). After the addition of **3-meta** was finished, the reaction mixture was stirred for another 4 h, gradually reaching 0 °C. The reaction mixture was concentrated to dryness and directly separated by silica gel column chromatography, isocratically eluting with a ternary mixture of cyclohexane/ethyl acetate/acetonitrile 7/3/2. Concentration of the product-containing fractions provided the title material **5-meta** (=**TR2**) as a white crystalline solid (315 mg, 38% yield).

The Togni cross-linking reagent **TR1**, para-disubstituted analogue of **5-meta**, was prepared using the same overall synthetic sequence as Togni cross-linking reagent **TR2** differing only in the starting dihydroxybenzene scaffold (hydroquinone instead of resorcinol) and in minor reaction conditions and purification details: The analogous fluoroalkylation of hydroquinone with Halon 2402 also produced a tarry reaction mixture (Figure S5); after aqueous workup and vacuum distillation, an impure fraction of the bis-fluoroalkyl bromide **2-para** contaminated with the brominated side product and other byproducts was obtained. Compared to the resorcinol series, however, pure bis-fluoroalkyl bromide **2-para** could be isolated by column chromatography in 23% isolated yield. Conversion of bis-fluoroalkyl bromide **2-para** to the corresponding fluoroalkyl silane **3-para** (Figure S6) was achieved under Barbier conditions using trimethylsilyl chloride and the Turbo–Grignard reagent, affording bis-fluoroalkyl silane **3-para** in 85% yield and ∼90% purity, with the remaining impurity assigned to the partially protodesilylated material. Finally, Umpolung of the fluoroalkyl silane **3-para** was carried out with fluoroiodane acceptor **4** in the presence of tetrabutylammonium triphenyldifluoridosilicate (TBAT), providing the desired Togni cross-linking reagent **TR1** (=**5-para**) in 37% isolated yield as an off-white solid (Figure S7).

Reactions with air-sensitive materials were carried out under an argon atmosphere using standard Schlenk techniques. All solvents were dried by activated molecular sieves (3 Å) and stored under argon. All commercially available chemicals were used as received unless stated otherwise. Flash column chromatography was performed using silica gel 60 (0.040–0.063 mm) supplied by Fluorochem. TLC analyses were done using TLC silica gel 60 F254 glass plates from Rushan Sanpont, which were visualized under UV (254/366 nm) or using the potassium permanganate stain solution.

^1^H, ^13^C, and ^19^F NMR spectra of synthesis products (Figures S8–S21) were measured at ambient temperature using 5 mm diameter NMR tubes. ^13^C spectra were proton-decoupled. High-resolution MS spectra were recorded on solariX XR (15T, Bruker Daltonics, USA, Figure S22). Both Togni cross-linking reagents were analyzed in positive mode with one million transient data points over the range of 200–2500 *m*/*z*. The final spectrum was created by averaging eight spectra with accumulation ions in the collision cell for 0.5 s.

### Protein Preparation for Cross-Linking Reactions

Lyophilized horse heart MYO was dissolved in the labeling buffer (degassed 50 mM ammonium bicarbonate, pH 7.4) and purified using a gel chromatography column (ENrichTM SEC 70, Bio-Rad, USA). RHOA protein was expressed in *Escherichia coli* BL21 cells as described previously^27,28^ and purified using a gel chromatography column (ENrichTM SEC 70, Bio-Rad, USA). The final concentration of all proteins was determined by a UV–vis spectrophotometer (DeNovix DS-11). The preparation of apoMYO was carried out based on the protocol reported earlier.^24^ The concentration of both apo- and holoMYO was adjusted to 12 μM.

### Chemical Cross-Linking with Togni Cross-Linking Reagents

Both Togni cross-linking reagents (**TR1**, **TR2**) were freshly dissolved in tetrahydrofuran to a concentration of 40 mg/mL. When used, ascorbic acid was dissolved in the labeling buffer employed for protein cross-linking at a concentration of 10 mg/mL and served as the reaction initiator. The pH of the ascorbic acid stock solution was adjusted to 7.0–7.5 to maintain a stable pH during cross-linking experiments. Tryptophan, dissolved in the reaction buffer at a concentration of 49 mM, was used to quench the reaction. Methionine amide hydrochloride was added to each cross-linking reaction at a final concentration of 20 mM as an antioxidant. The pH of the methionine amide stock solution was adjusted to 7.0–7.5 prior to addition to the samples. All cross-linking reactions were performed in triplicate.

MYO cross-linking: Cross-linking of both apo- and holoMYO (12 μM, 50 μL) was carried out in labeling buffer. The cross-linking reagent was added to the sample to a final concentration of 2.4 mM. After the addition of the cross-linker, the samples were mixed, and ascorbic acid was added to the mixture to initiate the reaction (final concentration 1.7 mM). The cross-linking reactions were incubated at room temperature and quenched after 5 min by addition of the quenching solution to a final concentration of 24.5 mM.

RHOA cross-linking: Chemical cross-linking of the RHOA (8 μM, 50 μL) protein was conducted in 20 mM 4-(2-hydroxyethyl)-1-piperazineethanesulfonic acid (HEPES), 5 mM MgCl_2_, 0.17 mM *n*-dodecyl β-d-maltoside (DDM), 5 mM tris­(2-carboxyethyl)­phosphine (TCEP) (pH 7.4). The Togni cross-linking reagent **TR2** was added to the sample to a final concentration of 1.6 mM; the reaction was allowed to proceed for 3 h at 37 °C. In the case of activation using ascorbic acid, the activator was added to the sample to a final concentration of 1.2 mM and incubated at room temperature for 5 min. In both approaches, the reaction was quenched by addition of the quenching solution to a final concentration of 24.5 mM. After the addition of the quenching solution, TCEP was added to every sample to a final concentration of 10 mM and incubated at room temperature for 20 min. Afterward, 2-iodoacetamide was added to the samples to a final concentration of 20 mM and incubated at room temperature for 20 min in the dark.

### Intact Mass Measurements

Intact mass measurements of modified apoMYO were used to determine and compare the reactivities of the two reagents used in this work. After quenching the reaction, samples were desalted using MicroTrap (Optimize Technologies, USA) and diluted 25 times into 50% methanol and 0.1% formic acid. Diluted samples were analyzed using an electrospray ionization coupled with a solariX FT-ICR mass spectrometer (Bruker Daltonics, USA) equipped with a 15T superconducting magnet. Data acquisition was performed using the following parameters: broadband mode with an *m*/*z* range from 250 to 2500 Th, an accumulation time of 0.2 s, and 32 averaged scans. In the case of spectra acquired with quadrupole isolation, the following parameters were used: an accumulation time of 1.5 s, 64 averaged scans, and Q1 *m*/z 960 ± 45 Da. Deconvolution was done using the SNAP algorithm implemented in the DataAnalysis 4.4 software with the following parameters: a quality factor of 0.8, a signal-to-noise ratio of 2, a relative intensity threshold of 5%, and a maximal charge state of 35.

### Digestion of Labeled Samples

After the cross-linking reaction, a volume of the sample corresponding to 5 μg of protein was transferred to a new microtube and diluted in a 1:1 ratio with the corresponding cross-linking buffer. Afterward, trypsin was added to every sample (protein/enzyme ratio 1:20) and the samples were incubated at 37 °C. After 4 h, trypsin was again added to every sample in a 1:20 ratio. The digestion was performed overnight at 37 °C.

### LC–MS/MS Analysis of Labeled Samples

The peptide mixture was diluted into 0.1% formic acid (FA), and 200 ng was injected into the Evosep One LC system (Evosep, Denmark) connected to the timsTOF SCP mass spectrometer (Bruker Daltonics, U.S.A.). The LC method used was the “30 samples per day” with a 44 min long gradient. Solutions A and B for the LC consisted of 0.1% FA and 0.1% FA in ACN, respectively. The MS acquisition method had the following parameters: mass spectra were acquired from 100 to 1700 *m*/*z* and 0.6 to 1.6 V·s·cm^–2^ in positive mode with a ramp time of 100 ms, ten PASEF scans in a cycle (total cycle time: 2.25 s), precursor charge from 2+ to 8+, isolation window 2 *m*/*z* for *m*/*z* ≤ 600 and 3 *m*/*z* for *m*/*z* ≥ 700, dynamic exclusion was enabled for 0.4 min (*m*/*z* width 0.015 and 1/k width 0.015 V·s·cm^–2^). The collision energy was set to 21.25 eV for 0.73 V·s·cm^–2^ and to 72.75 eV for 1.63 V·s·cm^–2^ and linearly interpolated between these two values.

### Identification of Cross-Links

The acquired raw data from the bottom-up experiment were processed and exported to a file in the Mascot Generic Format by the DataAnalysis 5.3 software using a self-written script.^29^ For the identification of cross-links, MeroX 2.0 software was used with the following parameters: lysine and arginine residues were set as protease sites with three missed cleavages allowed. Methionine oxidation and carbamidomethylation of cysteine residues were set as variable modifications (up to three modifications per peptide were allowed). Togni cross-linking reagent composition was set as follows: C_10_F_8_O_2_H_2_ (305.993) in case of the main product and C_10_F_6_O_3_H_2_ (283.991) in case of the side product, site 1 and 2 specificities were set to phenylalanine, histidine, tryptophan, tyrosine, and cysteine for the main product; site 1 was set to phenylalanine, histidine, tryptophan, tyrosine, and cysteine and site 2 to lysine, threonine, serine, tyrosine, and N-termini for the side product. Type 0 modifications were set with the following compositions: C_16_F_8_O_8_H_10_ (482.025) in the case of type 0, C_10_F_6_O_4_H_4_ (302.001) in the case of type S0a, C_10_F_6_O_3_H_5_N_1_ (301.017) in the case of type S0b, and C_16_F_6_O_9_H_10_ (460.023) in the case of type S0c. MS1 precision was specified to 15 ppm and minimal charge of precursor to 3, and MS2 precision to 25 ppm with a lower mass limit of 200 Da and an upper mass limit of 8000 Da. The signal-to-noise ratio was set to 2. Only b and y fragments were considered. Quadratic mode was used. For the scoring and FDR settings, the prescore was set to 20%, the FDR cutoff to 5%, and the score cutoff to 15. Shuffled sequences (protease sites were kept) were used for the generation of the decoy database. All identified products were checked manually. Relevant results from triplicate measurements were plotted into the X-ray structure and visualized by software PyMol 3.2.0a (Schrödinger, USA). Additionally, type 2 products were visualized using xiVIEW.^30^

## Results and Discussion

### Synthesis of Togni Cross-Linking Reagents

The synthetic sequence of both Togni cross-linking reagents was based on a published synthetic strategy relying on Umpolung of an appropriate fluoroalkyl silane with a hypervalent iodine-fluoride precursor.^19^ To construct the prerequisite bis-fluoroalkyl silane **3-meta** based on a 1,3-dihydroxybenzene scaffold, first we attempted to convert resorcinol **1-meta** into the corresponding resorcinol-bis-tetrafluoroethyl bromide **2-meta**. Resorcinol **1-meta** was deprotonated with sodium hydride in anhydrous dimethylformamide and reacted with an excess of Halon 2402 (1,2-dibromo-1,1,2,2-tetrafluoroethane). Unfortunately, the reaction did not have a clean course, resulting in a tarry reaction mixture from which the desired product could be isolated as an inseparable mixture of **2-meta** with its brominated analogue **2-Br-meta** in a low yield of 25% (2:1 mixture) by vacuum distillation of the extract. The likely explanation is that double deprotonation renders resorcinol **1-meta** (or its dianion, respectively) extremely vulnerable to electrophilic bromination by Halon 2402, leading to ring bromination and also oxidative degradation, producing tarry side products. To circumvent this issue, we employed a gradual release of the monoTMS-resorcinol anion through in situ deprotection of resorcinol-bis-trimethylsilyl ether **1-TMS** in the presence of alkali metal fluoride. During the course of deprotection, the monoanion generated during the process would be significantly less electron-rich than the dianion, rendering it much less susceptible to ring bromination and oxidative degradation. Resorcinol was subjected to double silylation by reacting with an excess of hexamethyldisilazane in acetonitrile in the presence of catalytic amounts of saccharin in quantitative yield. Resorcinol bis-trimethylsilyl ether **1-TMS-meta** was then subjected to fluoroalkylation in the presence of either spray-dried potassium fluoride or cesium fluoride in DMF. While the use of spray-dried KF appeared to provide the product with less than 5% of the brominated impurity, the product yield of the overnight reaction was low (15%), and the reaction was incomplete, suggesting slow desilylation rates. Switching to the more reactive cesium fluoride raised the isolated yield to 67%, and the brominated side product was present as a 3–5% impurity. With still incomplete suppression of the bromination side reaction, we turned our attention to further improving the course of fluoroalkylation and eliminating this side reaction. We speculated that in the initial phases of fluoroalkylation, Halon 2402 needs to release tetrafluoroethylene by bromophilic attack of the nucleophile, and this is the likely reason for the observed minor ring bromination. We expected that addition of external tetrafluoroethylene gas from the very beginning of the reaction limits the need for the initial halophilic attack on Halon 2402, thus providing a cleaner reaction course. Indeed, running the same reaction under a TFE atmosphere supplied from a balloon provided the desired bis-fluoroalkyl bromide **2-meta** in 75% isolated yield as a clear, slightly yellowish oil. Subsequent conversion of the bis-fluoroalkyl bromide **2-meta** to the corresponding bis-fluoroalkyl silane **3-meta** was achieved by treating the bis-fluoroalkyl bromide **2-meta** under Barbier conditions with TMSCl and Turbo-Grignard solution, leading to isolation of bis-fluoroalkyl silane **3-meta** as a clear oil in 85% isolated yield with a trace of partially protodesilylated material. The last step of the sequence was the Umpolung of bis-fluoroalkyl silane **3-meta** with cyclic hypervalent iodine-fluoride precursor **4** in the presence of TBAT as a catalyst. The desired bis-Togni reagent **TR2** (**5-meta**) was isolated by column chromatography in 38% isolated yield as a white crystalline powder. The Togni cross-linking reagent **TR1** was prepared following the same synthetic sequence with few differences described in the Experimental Section, and the target compound **TR1** was obtained in 37% isolated yield. **TR1** and **TR2** were prepared as a minimal regioisomeric pair to assess whether the meta- and para-substitution patterns of the central aromatic scaffold affect the effective orientation of the reactive termini and, consequently, productive cross-link formation. This regioisomeric comparison is also relevant for the future development of isotopically encoded analogues, for which the synthetic strategy is expected to depend on the substitution pattern of the central scaffold.

### Chemical Cross-Linking Reaction

Since it has been shown that Togni reagents form a covalent bond with aromatic amino acid side chains within the protein sequence in the presence of ascorbate and can also react with cysteine sulfhydryl without any external activation,^24^ MYO and RHOA have been selected as model proteins. As shown in Figure 3, the cross-linking reaction with **TR1** and **TR2** could lead to the formation of several products: (a) type 1 or type 2 product between either two aromatic residues or one aromatic residue and one cysteine residue or two cysteine residues; (b) formation of type 0 product corresponding to the formation of a bond between either aromatic residue or cysteine on one side of the reagent and ascorbate on the other side of the reagent.

**Figure 3:**
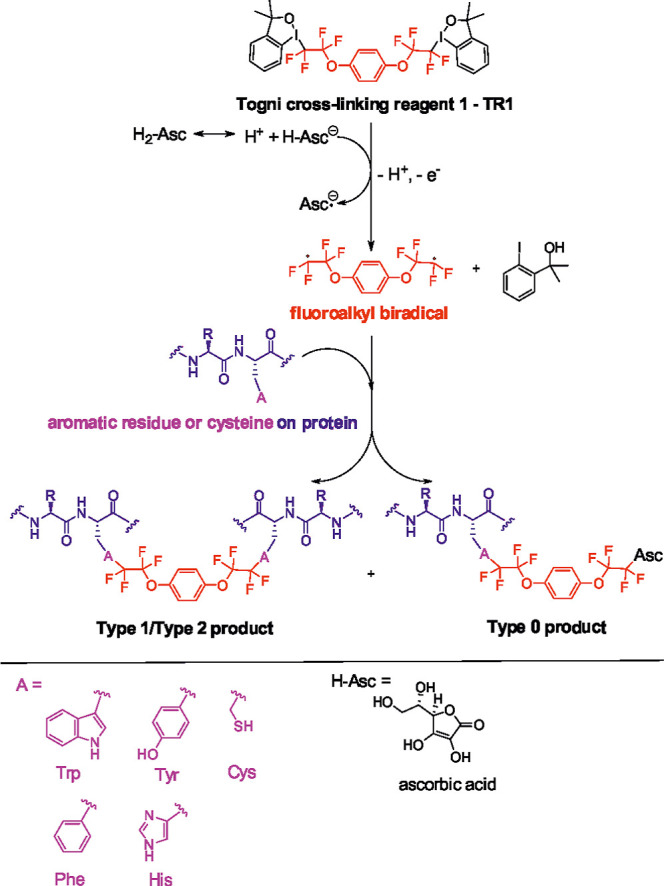
Reaction scheme of the cross-linking reaction with Togni cross-linking reagent 1 (**TR1**) using FFAP. Ascorbic acid induces the generation of a fluoroalkyl biradical from **TR1**. The resulting fluoroalkyl radical reacts with aromatic residues or cysteine, generating type 1/type 2 and type 0 products. TR2 follows the same reaction scheme.

Apo- and holoMYO served as model proteins to benchmark the reactivity of the new reagents because both forms are structurally well characterized (X-ray crystallography,^31^ HDX,^32^ and FPOP^33^) and MYO was shown to be efficiently labeled with Togni reagents under the FFAP protocol developed in our group.^21^ Moreover, the MYO sequence contains a high abundance of aromatic residues that are the sites of modification when utilizing the FFAP.

We used intact-protein mass spectrometry of the modified samples to assess the reactivity of the new probes. Both Togni cross-linking reagents **TR1** and **TR2** successfully modified apoMYO. The modification extent when using reagent **TR1** was significantly lower than the extent of modification when using reagent **TR2** (Figure 4). In addition to the unmodified protein, the mass spectra predominantly showed ions corresponding to either a single type 1 or 2 product or a singly modified species consistent with the type 0 product, suggesting either limited reactivity and/or modification at preferred residues. The observation of the type 0 product is consistent with the proposed dual role of ascorbic acid in the FFAP workflow,^34^ acting not only as a reaction initiator but also as a radical scavenger. We also detected a species exhibiting a mass shift of −22 Da relative to the cross-linked product, suggesting possible substitution of two fluorine atoms by one oxygen atom (isotopic pattern labeled with an asterisk in Figure 4) that was described previously.^35^ The proposed mechanism for the formation of this side product of the reaction is shown in Figures S23 and S24.

**Figure 4:**
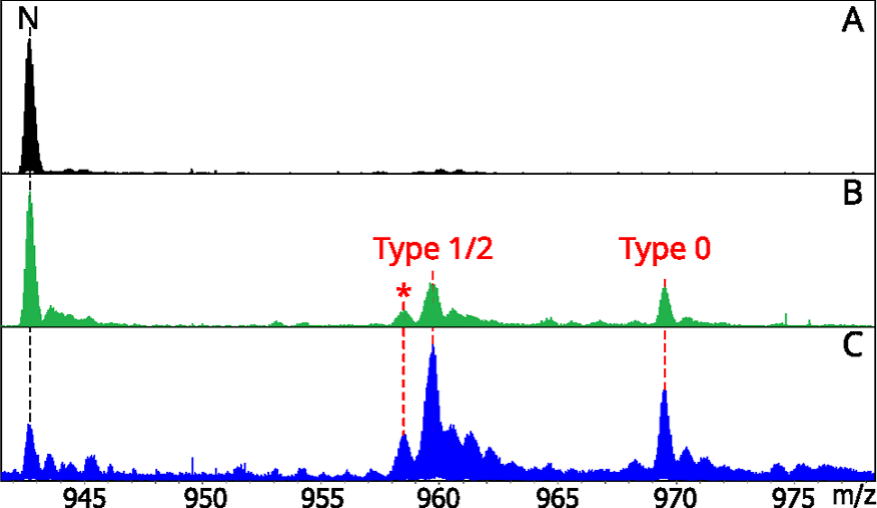
Mass spectra of the 18^+^ charge state of apoMYO: control (A) and after the reaction with Togni cross-linking reagent **TR1** (B) and Togni cross-linking reagent **TR2** (C); isotopic pattern of the nonmodified protein is denoted as N, type 1 and type 2 products as type 1/2, and type 0 product as type 0. Asterisk indicates modified protein with type 1/2 product and mass shift −22 Da.

To identify the modified residues in apoMYO, bottom-up analysis of the modified protein was performed. In total, two (H64–H93 and H93–F138) and four (F43–H64, F43–H93, H64–H93, and H93–F138) type 2 products were identified in samples treated with Togni cross-linking reagents **TR1** and **TR2**, respectively (Tables S1 and S2 and Figure 5). Notably, H93 was especially susceptible to modification as it participated in multiple cross-links. All type 2 products, irrespective of the reagent used, involved aromatic residues oriented toward the heme-binding cavity (e.g., H93). This suggests that the reagents preferentially react with residues that mediate heme interactions and/or are in the vicinity of the heme in holoMYO. However, the distribution of modification reflects the local protein microenvironment of apoMYO, where the empty hydrophobic heme-binding cavity may promote productive placement of reactive termini. The observed clustering of products around the heme-binding region should be interpreted as a feature of this particular protein–reagent system rather than as a general restriction of the chemistry to heme-pocket residues. Consistent with this interpretation, the crystal structure (PDB: 1WLA, L104N variant)^31^ shows that H93 coordinates the ferrous iron in the center of the heme, while the remaining cross-linked aromatic residues cluster around the heme moiety. All of the identified type 2 products aligned well with the crystal structure of MYO and show the preference of the reagent toward residues in the heme cavity (Figure 5).

**Figure 5:**
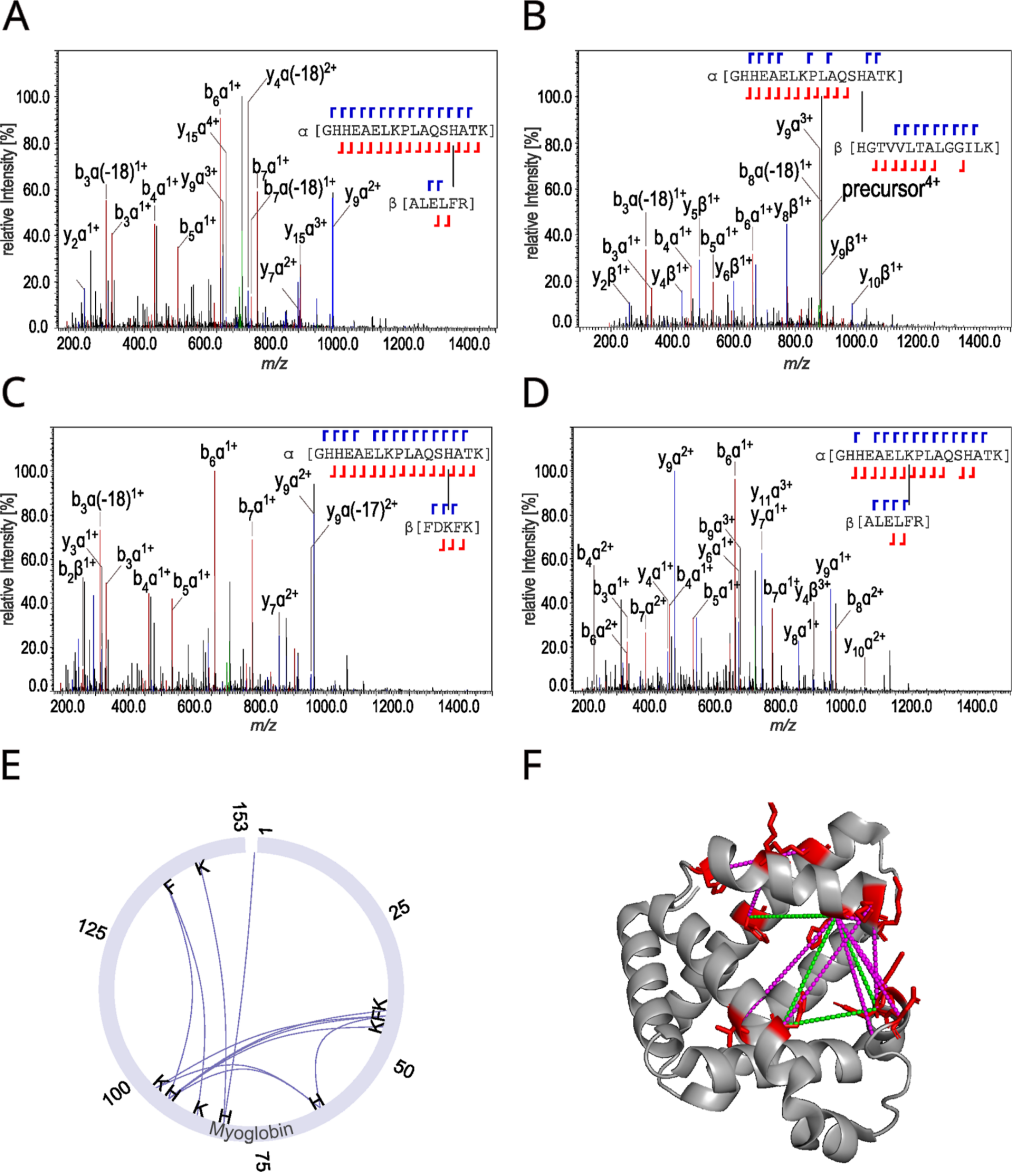
Examples of identified type 2 products of apoMYO upon reaction with Togni cross-linking reagent **TR2** by MeroX software, specifically H93–H64 (A), H93–F138 (B), H93–K45 (C), and K96–F138 (D). B fragments are highlighted in red, y fragments in blue, and the precursor in green. Sequence map of apoMYO with identified type 2 products (E). Crystal structure of horse heart holoMYO (1WLA) without heme for visualization of the apo-like heme binding cavity and highlighted type 2 products (F); main reaction products indicated in green and side products indicated in magenta.

Regarding type 0 products, five (F33, H36, H82, H93, and H119) and eight (H24, F33, H36, F43, H82, H93, H97, and H119) were identified for cross-Togni linking reagents **TR1** and **TR2** (Tables S1 and S2 and Figures S25 and S26), respectively, with a higher number of modified residues observed for reagent **TR2**. Similar to the type 2 products, most type 0 products were situated in proximity to the heme-binding site in the crystal structure of holoMYO.

Furthermore, we also searched the bottom-up data of apoMYO for the modification with a loss of 22 Da. On the basis of the literature,^35^ we speculated that it would correspond to an aromatic residue connected to a nucleophilic group (e.g., primary amine, alcohol) and thus the formation of a type 2 product. This hypothesis was confirmed by the search results. In total, three type 1 products (K42–F43, H82–K87, K96–H97) and four type 2 products (K42–H64, H81–K98, K45–H93, and H93–K145) were identified using **TR1** (Table S3), whereas three type 1 products (K42–F43, H82–K87, H93–K96) and eight type 2 products (K42–H93, F43–K96, K45–H93, H64–K96, T66-H93, H82–K145, K87–F138, and H93–K98) were identified using Togni cross-linking reagent **TR2** (Table S4). As with the main products of the cross-linking reaction, the identified type 2 products aligned well with the crystal structure of MYO and modified mainly residues close to the heme cavity (Figure 5).

The oxygenated side product was not observed in our previous study using monofunctional Togni reagents and was, therefore, not reported. We speculate that its formation depends on the specific reagent architecture, particularly on the composition of the fluoroalkyl chain released upon activation.

In apoMYO, we also detected two type 1 products (K63-T66 and S92-T95) for **TR1** and one type 2 for **TR2** (T39-S92), indicating that cross-link formation between two nucleophilic groups can occur but represents a minor reaction pathway under the conditions employed (Tables S3 and S4). We further identified three different species of side type 0 product (labeled as type S0a–c) that arise due to the formation of acyl fluoride intermediates (Figures S23 and S24). These involve aromatic or sulfhydryl side chains modified by fluoroalkyl radicals formed on one end of the cross-linker and formation of acyl fluoride on the other end that reacts with water molecule (a), with the ammonia molecule (b) and nucleophilic side chain modified by acyl fluoride formed on one end of the cross-linker and ascorbic acid that quenches the fluoroalkyl radical formed on the other side of the reagent (c). For **TR1**, nine type S0a (F33, H36, F43, K56, H82, H93, H119, F123, F138), five type S0b (F33, H64, H82, H93, H119), and seven type S0c (K42, K77, K82, K87, H93, K96, K98) were identified in apoMYO (Table S5), whereas ten type S0a (H24, F33, H36, F43, K56, H64, H82, H93, H97, H119), six type S0b (H24, F33, H36, F43, H93, H119), and eight type S0c (K42, K56, K79, H82, K87, H93, K96, K98) were identified in apoMYO using **TR2** (Table S6).

To further test the idea that the new reagents preferentially modify residues in the vicinity of the heme, we modified holoMYO with both reagents and analyzed the samples using a bottom-up approach. On the basis of the data analysis of apoMYO, we speculated that the heme would interfere with the cross-linking reaction. Consistent with this hypothesis, no type 2 products were found, supporting the notion that the heme would suppress or prevent formation of these products. Instead, only four type 0 products were found in the samples modified with both Togni cross-linking reagents **TR1** and **TR2** (both reagents modified H24, F33, F43, and H119, Tables S7 and S8). When compared with the results of apoMYO labeling, we specifically noted the absence of H82 and H93 (located in two consecutive tryptic peptides) in the modified residues of holoMYO. Taking into account the fact that H93 facilitates the coordination of the heme moiety,^31^ these findings suggest that the empty heme cavity is required for the modification to occur to an observable extent and thus support the idea that the heme would interfere with the reaction presumably by providing steric hindrance.

We further identified several side products of the cross-linking reaction on holo-MYO. Specifically, one (K42–F43) and two (K42–F43 and F43–K45) type 1 products were obtained using Togni cross-linking reagents **TR1** and **TR2**, respectively (Tables S9 and S10). We also identified one type 2 product (W14–K77) using reagent **TR2** (Table S10). In addition, seven type S0a (H24, F33, H36, K56, H119, F123, F138), three type S0b (H24, F33, H119), and five type S0c (K42, K50, K56, K77, K87) were found in holoMYO samples using Togni cross-linking reagent **TR1**, whereas one type S0b (H119) and four type S0c (K42, K50, K77, K87) were identified using Togni cross-linking reagent **TR2** (Tables S11 and S12).

We speculate that the overall lower amount of either type of product in holoMYO compared with apoMYO could be attributed to the hydrophobicity of the reagent that made the transition of the reagent from the solution into the heme cavity favorable. As noted above, all obtained cross-links in apoMYO span the heme cavity, and even most of the residues modified with type 0 products are situated in the vicinity of the heme cavity. These results are in contrast to the results of cross-linking using conventional cross-linking reagents (e.g., NHS esters), which typically modify and/or connect solvent-exposed residues on protein surfaces, further suggesting different behavior of reagents **TR1** and **TR2** from the well-established cross-linking reagents.

Although we identified a higher number of side products compared to the expected ones, based on the intact mass measurements, their overall abundance in the sample is lower (Figure 4). We speculate that the higher number of these products reflects at least in part the higher conformational flexibility of the lysine side chains (out of the nucleophilic side chain-containing amino acids, but lysines were the ones mainly modified in the experiment) compared to the rigidity of the aromatic rings of phenylalanine and histidine. Furthermore, we should consider the abundance of lysine residues (19 in the MYO sequence).

We hypothesize that the above-mentioned factors contribute to the higher number of favorable side chain combinations that can form a cross-link bond and thus an overall higher number of identified unique combinations. This hypothesis is supported by the observation that only one additional aromatic residue (H82) participated in the unexpected type 2 product formation, while all other aromatic residues (F43, H64, H93, and F138) were also found in the main product.

To further explore the reactivity of the newly synthesized reagent, we used RHOA as a model protein. Due to the hydrophobicity of the reagents, we hypothesized that choosing a hydrophobic protein might lead to identification of a high number of products (regardless of their type). For this experiment, we used only Togni cross-linking reagent **TR2**, as this reagent was more efficient, well documented by the intact mass measurement (Figure 4) and bottom-up data analysis (Tables S1 and S2). Interestingly, out of all the aromatic residues in the sequence of RHOA, only one (F176) type 0 product was identified. Furthermore, three type 0 products (C21, C112, C195) and one type 2 product (C164–C195) were identified on the cysteine residues (Table S13).

Based on the above-described results and early published report,^22^ we decided to explore the possibility of using **TR2** without external activation–redox initiation using cysteine side chains. RHOA contains six cysteine residues in its sequence, and the fact that they do not form disulfide bonds makes this protein a suitable model system to test the activation of the reagent using cysteine side chains. Using this experimental approach, three type 2 products between cysteine residues (C112–C164, C112–C195, C164–C195) were identified, confirming the ability of cysteine residues to activate the reagent (Table S14). In addition, one type 2 product between a cysteine and tryptophan residue was detected, namely, C112–W63, further supporting that this activation mode can also yield type 2 products involving aromatic residues (Table S10), which is in line with a previously reported study on peptide fluoroalkylation.^36^ Since ascorbic acid was not used in the reaction, no type 0 products were identified. Mapping the obtained cross-links onto the available crystal structure (PDB: 5CM4)^37^ showed that the distance constraints between modified amino acids almost correspond to the length of the Togni cross-linking reagent arm (Figure 6). However, these distances were measured on a static X-ray structure, which may not fully represent the conformational ensemble present in solution under the cross-linking conditions used here. In addition, the available RHOA structure is missing or flexible segments which may affect the apparent distances for selected restraints.

**Figure 6:**
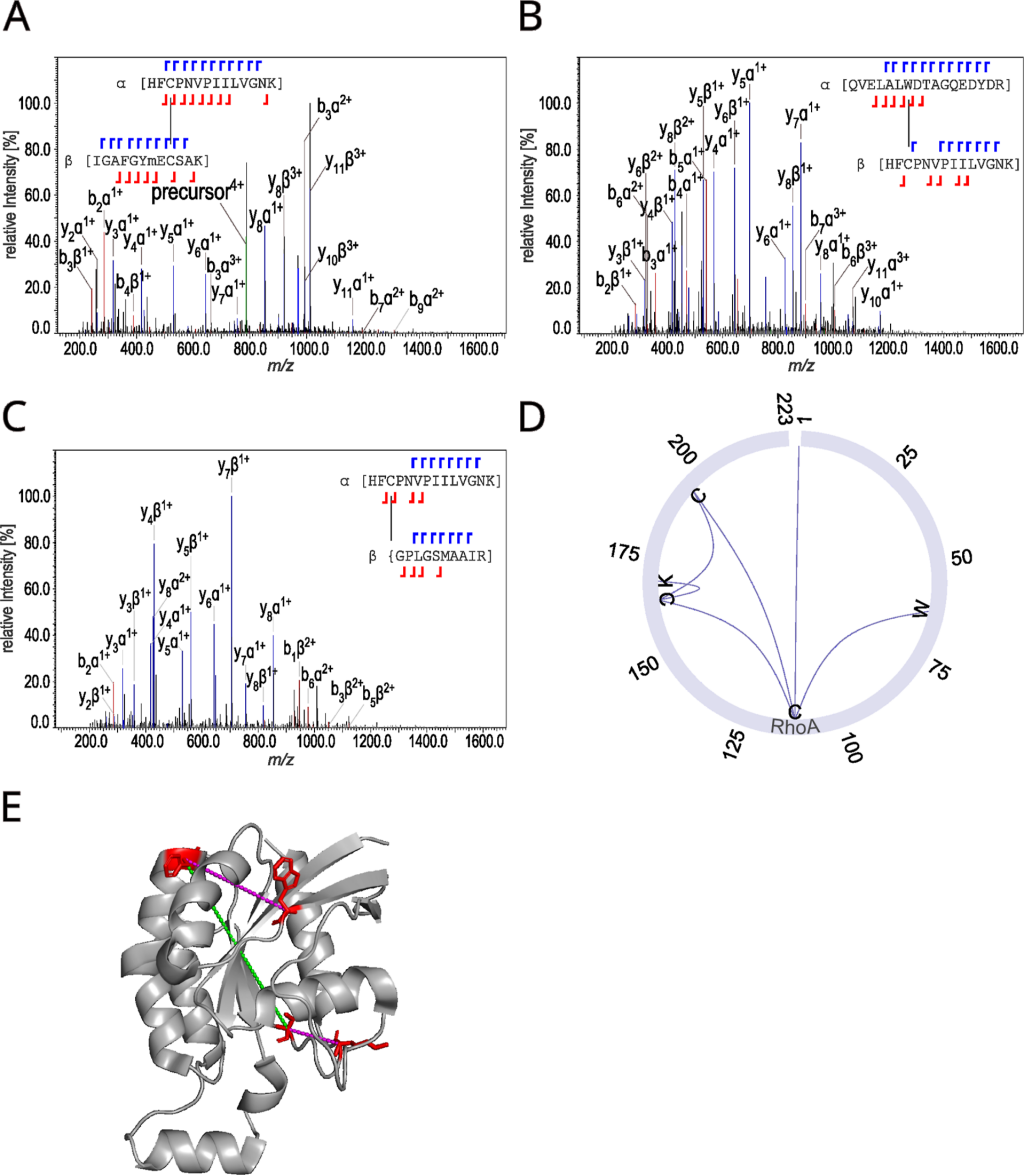
Examples of identified type 2 products of RHOA upon reaction with Togni cross-linking reagent **TR2** by MeroX software, specifically C112–C164 (A), W63–C112 (B), and C112–N-termini (C). B fragments are highlighted in red, y fragments in blue, and the precursor in green. Sequence map of RHOA with identified type 2 products (D). Crystal structure of RHOA (5C4M) with highlighted type 2 products (main products are shown in green, side products in magenta) (E).

Regarding the side products of the reaction, we identified one type 1 product (T105-C112), seven type S0a (C21, W63, W104, C112, C164, F176, C195), and two type S0c (G1, K169) when using ascorbate for the activation, while one type 1 product (T105-C112), two type 2 products (G0-C112, C164–K169), six type S0a (C21, W63, C112, C164, F176, C195), and two type S0b (C112, C195) were found in the experiment without external activation of the reagent (Tables S15–S18).

Although the RHOA sequence contains enough phenylalanine (8) and histidine (3) residues that were predominantly modified in MYO, only one of them was modified (type 0 product on F176) when using ascorbic acid as the initiator of the reaction. On the other hand, three cysteine residues (C21, C112, C195) were modified with type 0 modification, and two (C164, C195) were modified with type 2 product. Without external activation, the side chains of cysteine residues were the only sites of modification (three cysteine residues were modified this way—C112, C164, C195). In the case of RHOA, the data from both approaches thus suggest higher preference of the reagent for the thiol group of cysteine residues over the side chains of aromatic amino acids.

## Conclusion

In this work, we introduced two bifunctional hypervalent iodine reagents (Togni cross-linking reagents **TR1** and **TR2**) as a new class of radical cross-linkers for CXMS under mild aqueous conditions. Using apoMYO/holoMYO and the small GTPase RHOA as model systems, we demonstrate that these probes generate structurally informative restraints from type 2 products with preference for cysteine and aromatic residues. In MYO, the reaction is strongly shaped by the heme-binding pocket, yielding modifications that localize in and around the cavity in apoMYO and are markedly suppressed in holoMYO, consistent with the steric environment of probe access. Importantly, the probes can be activated either by ascorbate (FFAP protocol) or in the absence of an external reductant, through thiol-driven initiation on cysteine residues, enabling complementary labeling conditions. Beyond the expected products, we also observe additional type 2 products consistent with partial conversion of the fluoroalkyl motif to oxygenated species in aqueous buffers, which expands the attainable set of distance constraints by engaging nucleophilic side chains such as lysine. Together, these results establish bis-hypervalent iodine-fluoroalkyl chemistry as a versatile platform for generating orthogonal CXMS restraints and highlight amphibious Togni cross-linking reagents as practical additions to the structural proteomics toolbox.

## Supplementary Material



## Notes

Figures S1–S7: reaction schemes of preparation of reagents **TR1** and **TR2**; Figures S8–S21: NMR spectra of products and intermediates of Togni cross-linking reagent **TR1** and **TR2 s**ynthesis; Figure S22: MS spectra of **TR1** and **TR2**; Figures S23–S24: reaction schemes of the side cross-linking reaction with **TR1**; Figures S25–S26: structures of apoMYO with type 0 products for **TR1** and **TR2**; and Tables S1–S18: tables of all identified products of the cross-linking reaction of studied proteins with **TR1** and **TR2** including side products using FFAP or without external activation (PDF)

## Abbreviations

CXMS: chemical cross-linking mass spectrometry
FFAP: fast fluoroalkylation of proteins
FPOP: fast photo-oxidation of proteins
IAA: 2-iodacetamide
CryoEM: cryo-electron microscopy
NMR: nuclear magnetic resonance
NHS: *N*-hydroxysuccinimide
HDX: hydrogen/deuterium exchange
MS: mass spectrometry
apoMYO: apomyoglobin
holoMYO: holomyoglobin
HEPES: 4-(2-hydroxyethyl)-1-piperazineethanesulfonic acid
DDM: *n*-dodecyl β-d-maltoside
TCEP: tris­(2-carboxyethyl)­phosphine
TFA: trifluoracetic
DMF: dimethylformamide
THF: tetrahydrofuran
1-TMS meta: resorcinol bis-trimethylsilyl ether
