# Supplementary material for: An Amphibious Bifunctional Probe for Protein Chemical Cross-Linking
Source: J Am Chem Soc. 2026 Jul 8;148(28):29883–94. doi: 10.1021/jacs.6c04886 (PMC13397570; doi:10.1021/jacs.6c04886)
Supplement: Supplementary file 1 [file ja6c04886_si_001.pdf]

# Supplementary material

## An Amphibious Bi-functional Probe For Protein Chemical Cross-linking.

Michael Karpíšek[†,‡], Lukáš Fojtík[†,‡], Jan Fiala[†,‡], Vojtěch Langer[¶], Václav Matoušek[¶], Zdeněk Kukačka[*,†] and Petr Novák[*,†]

[†]Institute of Microbiology of the Czech Academy of Sciences, 14220 Prague, Czech Republic

[‡]Department of Biochemistry, Faculty of Science, Charles University, 12843 Prague, Czech Republic

[¶]CF Plus Chemicals, 62100 Brno, Czech Republic

### Table of contents


| | |
|---|---|
| 1, Reaction scheme of preparation of resorcinol bis-trimethyl silyl ether | S1 |
| 2, Reaction scheme of fluoroalkylation of resorcinol bis-trimethyl silyl ether | S2 |
| 3, Reaction scheme of conversion of bis-fluoroalkyl bromide to bis-fluoroalkyl silane | S3 |
| 4, Reaction scheme of Umpolung of bis-fluoroalkyl silane with fluoroidodane to Togni cross-linking reagent **TR2** | S4 |
| 5, Reaction scheme of preparation of hydroquinone bis-fluoroalkyl bromide | S5 |
| 6, Reaction scheme of conversion of bis-fluoroalkyl bromide to bis-fluoroalkyl silane | S6 |
| 7, Reaction scheme of Umpolung of bis-fluoroalkyl silane with fluoroiodane to Togni cross-linking reagent **TR1** | S7 |
| 8, $^{1}$H NMR Spectra of bis-fluoroalkyl bromide -compound 2-meta | S8 |
| 9, $^{19}$F NMR Spectra of bis-fluoroalkyl bromide - compound 2-meta | S9 |
| 10, $^{1}$H NMR spectrum of bis-fluoroalkyl silane - compound 3-meta | S10 |
| 11, $^{19}$F NMR spectrum of bis-fluoroalkyl silane - compound 3-meta | S11 |
| 12, $^{1}$H NMR Spectra of Togni cross-linking agent 2 - compound 5-meta | S12 |
| 13, $^{19}$F NMR Spectra of Togni cross-linking agent 2 - compound 5-meta | S13 |
| 14, $^{13}$C NMR Spectra of Togni cross-linking agent 2 compound 5-meta | S14 |
| 15, $^{1}$H NMR Spectra of bis-fluoroalkyl bromide -compound 2-meta | S15 |
| 16, $^{19}$F NMR Spectra of bis-fluoroalkyl bromide - compound 2-meta | S16 |
| 17, $^{1}$H NMR spectrum of bis-fluoroalkyl silane - compound 3-meta | S17 |
| 18, $^{19}$F NMR spectrum of bis-fluoroalkyl silane - compound 3-meta | S18 |
| 19, $^{1}$H NMR Spectra of Togni cross-linking agent **TR2** - compound 5-meta | S19 |
| 20, $^{19}$F NMR Spectra of Togni cross-linking agent **TR2** - compound 5-meta | S20 |
| 21, $^{13}$C NMR Spectra of Togni cross-linking agent **TR2** compound 5-meta | S21 |
| 22, MS spectra of Togni cross-linking reagent **TR1** and **TR2** | S22 |
| 23, Reaction scheme of side cross-linking reaction with **TR1** | S23 |
| 24, Reaction scheme of side cross-linking reaction with **TR1** | S24 |
| 25, Structure of apoMYO with Type 0 products for Togni cross-linking reagent **TR1** | S25 |
| 26, Structure of apoMYO with Type 0 products for Togni cross-linking reagent **TR2** | S26 |
| 27, Table of all identified products of cross-linking reaction of apoMYO with **TR1** | Table S1 |
| 28, Table of all identified products of cross-linking reaction of apoMYO with **TR2** | Table S2 |
| 29, Table of all identified side products of cross-linking reaction of apoMYO with **TR1** | Table S3 |

| | |
|---|---|
| 30, Table of all identified side products of cross-linking reaction of apoMYO with **TR2** | Table S4 |
| 31, Table of all identified Type 0 side products of cross-linking reaction of apoMYO with **TR1** | Table S5 |
| 32, Table of all identified Type 0 side products of cross-linking reaction of holoMYO with **TR1** | Table S6 |
| 33, Table of all identified products of cross-linking reaction of holoMYO with **TR1** | Table S7 |
| 34, Table of all identified products of cross-linking reaction of holoMYO with **TR2** | Table S8 |
| 35, Table of all identified side products of cross-linking reaction of holoMYO with **TR1** | Table S9 |
| 36, Table of all identified side products of cross-linking reaction of holoMYO with **TR2** | Table S10 |
| 37, Table of all identified Type 0 side products of cross-linking reaction of apoMYO with **TR2** | Table S11 |
| 38, Table of all identified Type 0 side products of cross-linking reaction of holoMYO with **TR2** | Table S12 |
| 39, Table of all identified products of cross-linking reaction of RHOA with **TR2** using FFAP | Table S13 |
| 40, Table of all identified products of cross-linking reaction of RHOA with **TR2** without external activation | Table S14 |
| 41, Table of all identified side products of cross-linking reaction of RHOA with **TR2** using FFAP | Table S15 |
| 42, Table of all identified side products of cross-linking reaction of RHOA with **TR2** without external activation | Table S16 |
| 43, Table of all identified Type 0 side products of cross-linking reaction of RHOA with **TR2** using FFAP | Table S17 |
| 44, Table of all identified Type 0 side products of cross-linking reaction of RHOA with **TR2** without external activation | Table S18 |

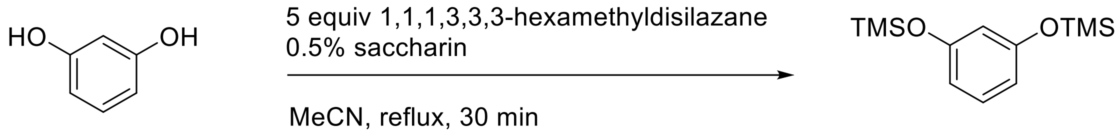


**1-meta** **1-TMS-meta**

**Figure S1:** Reaction scheme and condition for preparation of resorcinol bis-trimethyl silyl ether (compound **1-TMS-meta**) from resorcinol (compound **1-meta**)

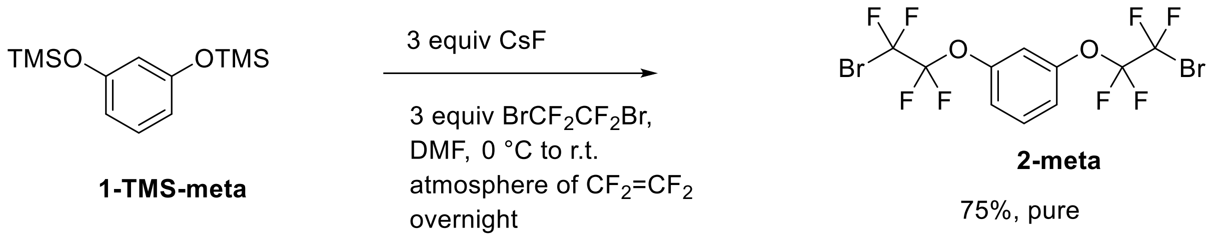


**1-TMS-meta** **2-meta**

75%, pure

**Figure S2:** Reaction scheme and condition for fluoroalkylation of resorcinol bis-trimethyl silyl ether (compound **1-TMS-meta**) to bis-fluoroalkyl bromide (compound **2-meta**)

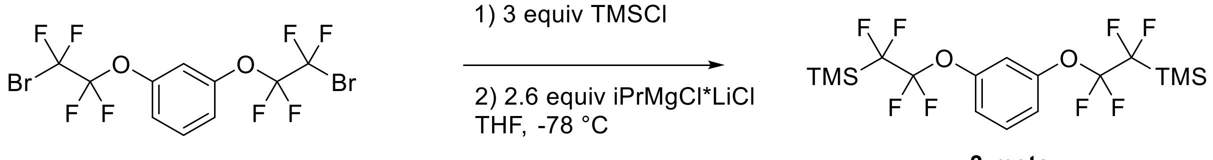


**2-meta** **3-meta**

85%

**Figure S3:** Reaction scheme and condition for conversion of bis-fluoroalkyl bromide (compound **2-meta**) to bis-fluoroalkyl silane (compound **3-meta**)

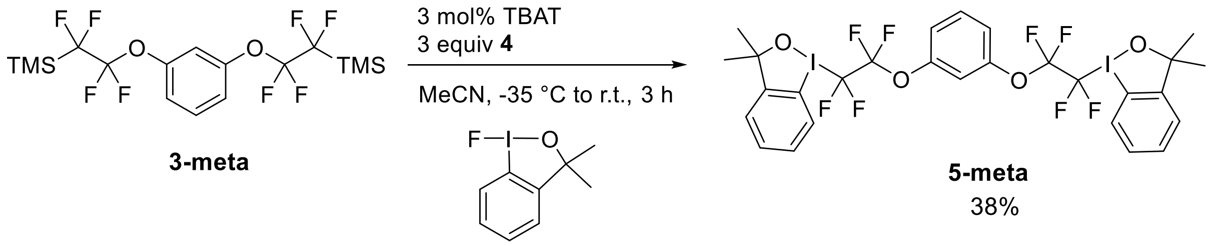


**3-meta** **5-meta**

38%

**Figure S4:** Reaction scheme and condition for Umpolung of bis-fluoroalkyl silane (compound **3-meta**) with fluoroidodane (compound **4**) to Togni cross-linking agent **TR2** (compound **5-meta**)

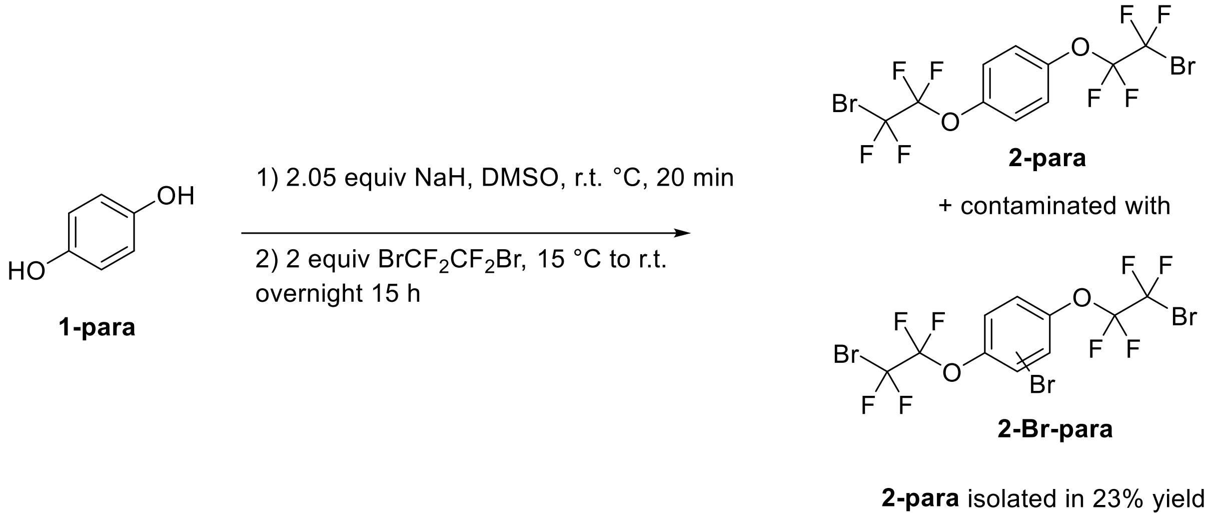


**Figure S5:** Reaction scheme and condition for preparation of hydroquinone bis-fluoroalkyl bromide (compound **2-para**) from hydroquinone (compound **1-para**)

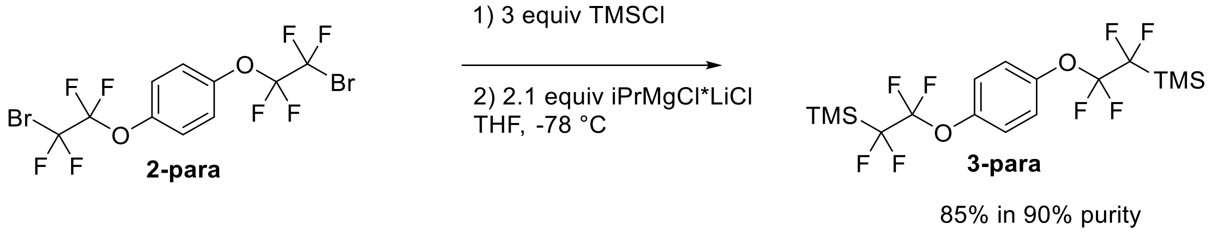


**Figure S6:** Reaction scheme and condition for conversion of bis-fluoroalkyl bromide (compound **2-para**) to bis-fluoroalkyl silane (compound **3-para**)

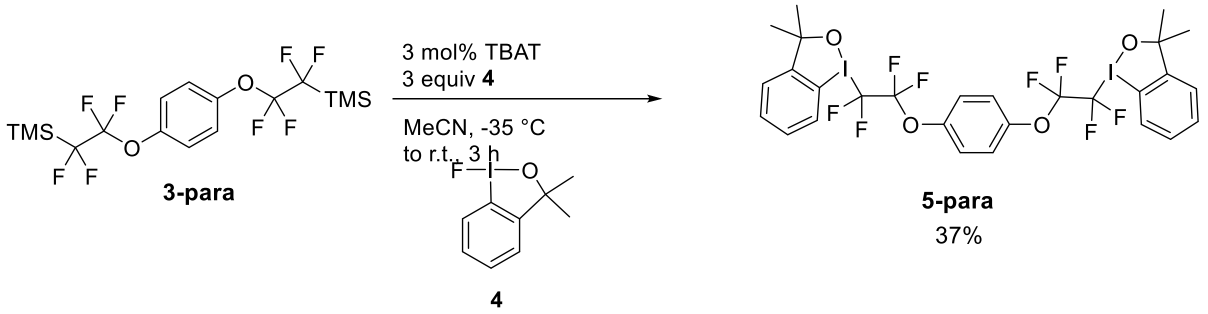


**Figure S7:** Reaction scheme and condition for Umpolung of bis-fluoroalkyl silane (compound **3-para**) with fluoroidodane (compound **4**) to Togni cross-linking agent **TR1** (compound **5-para**)

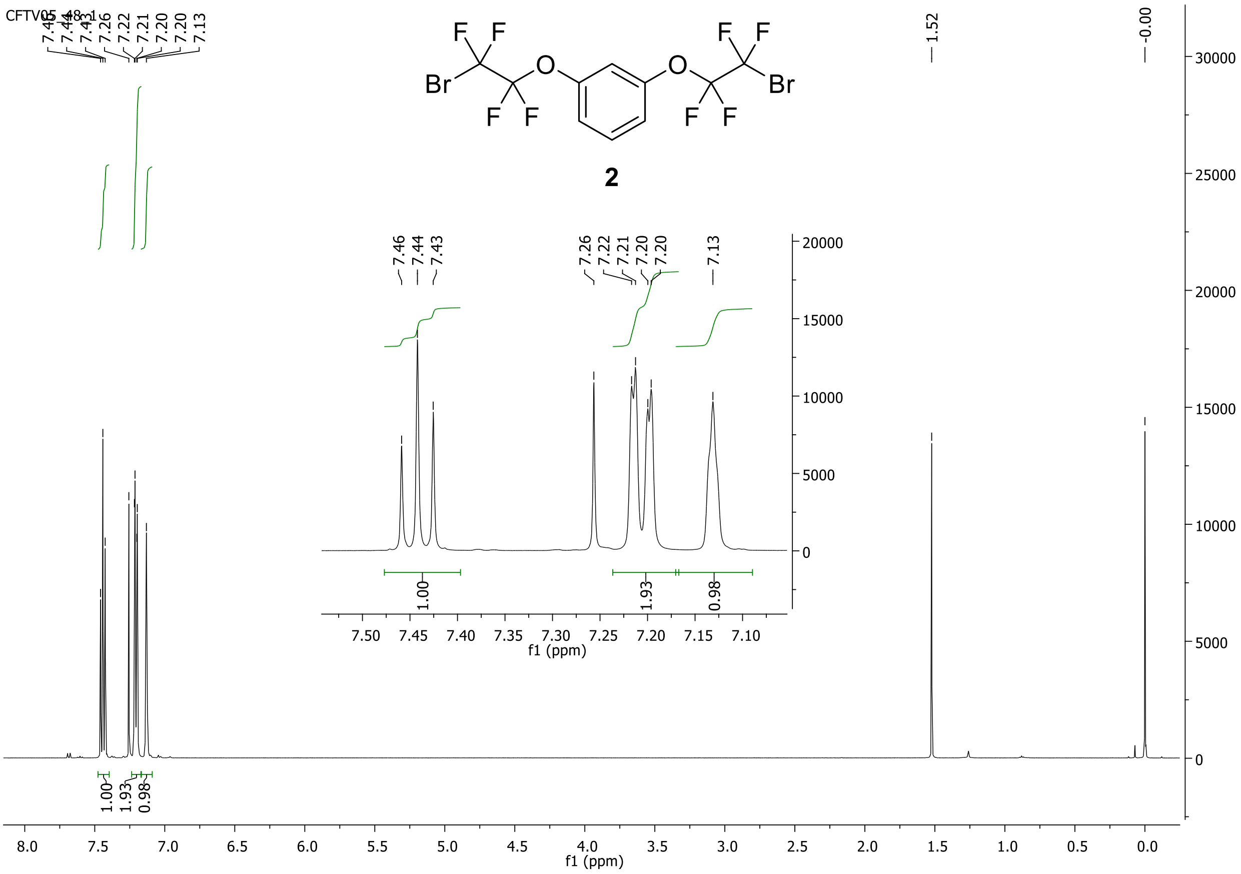


**Figure S8:** $^{1}$H NMR spectrum of bis-fluoroalkyl bromide (compound **2-meta**)

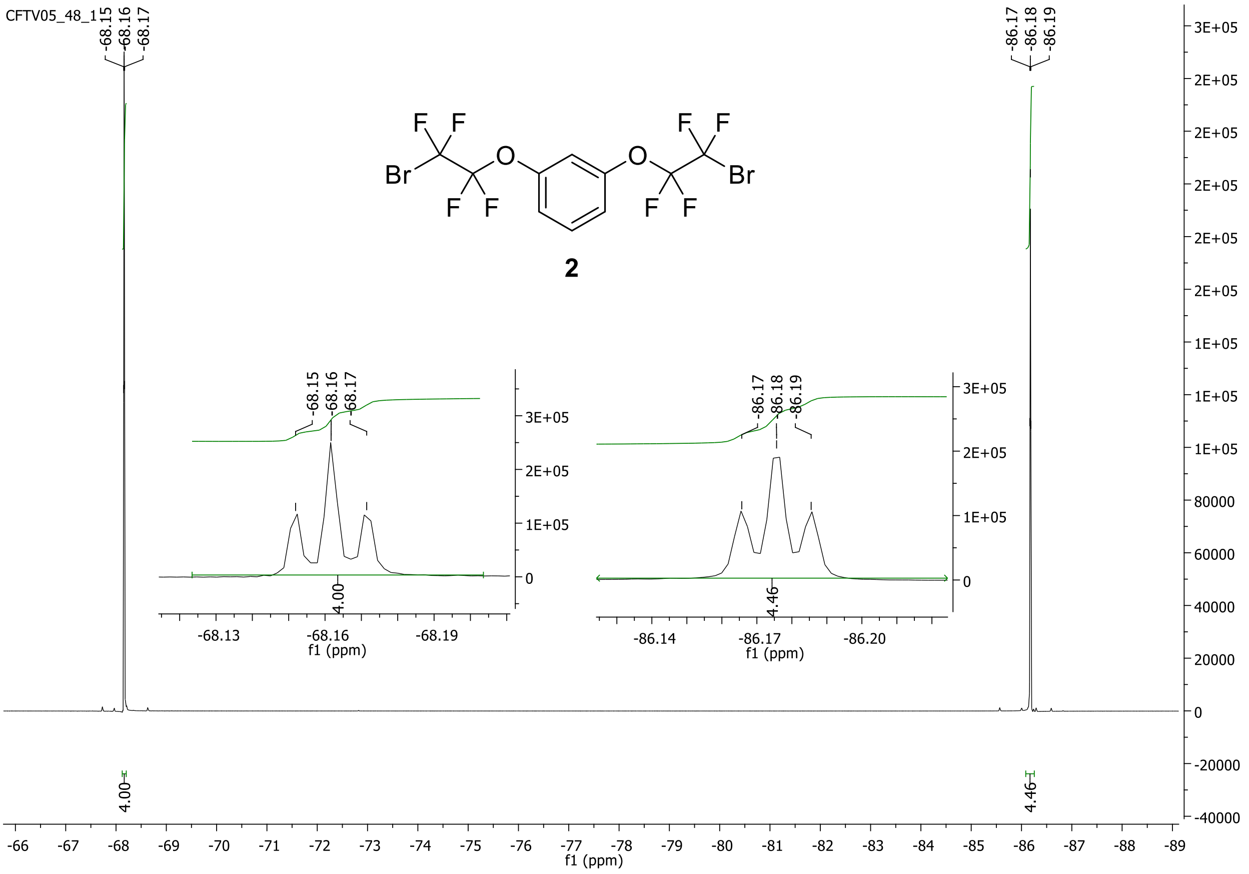


**Figure S9:** $^{19}$F NMR spectrum of bis-fluoroalkyl bromide (compound **2-meta**)

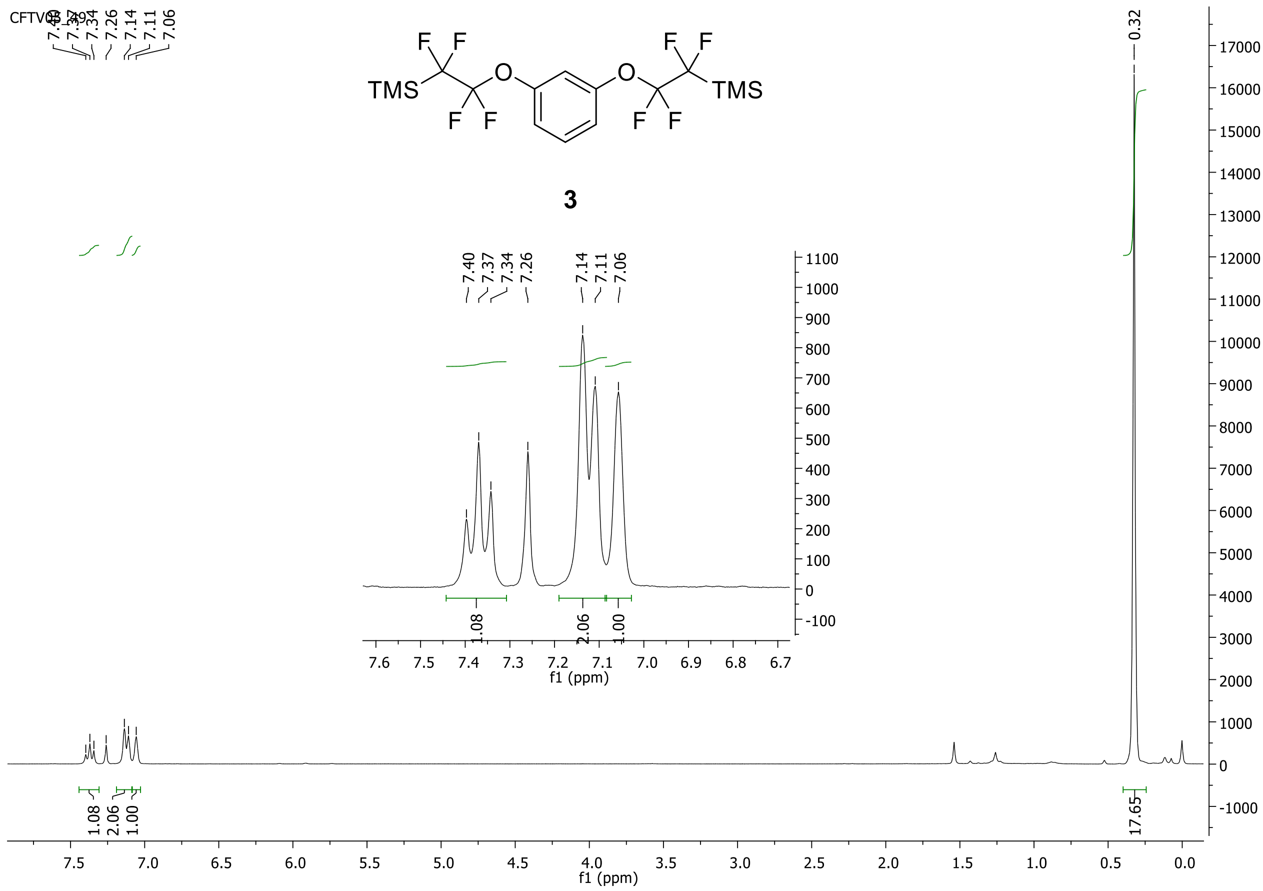


**Figure S10:** $^{1}$H NMR spectrum of bis-fluoroalkyl silane (compound **3-meta)**

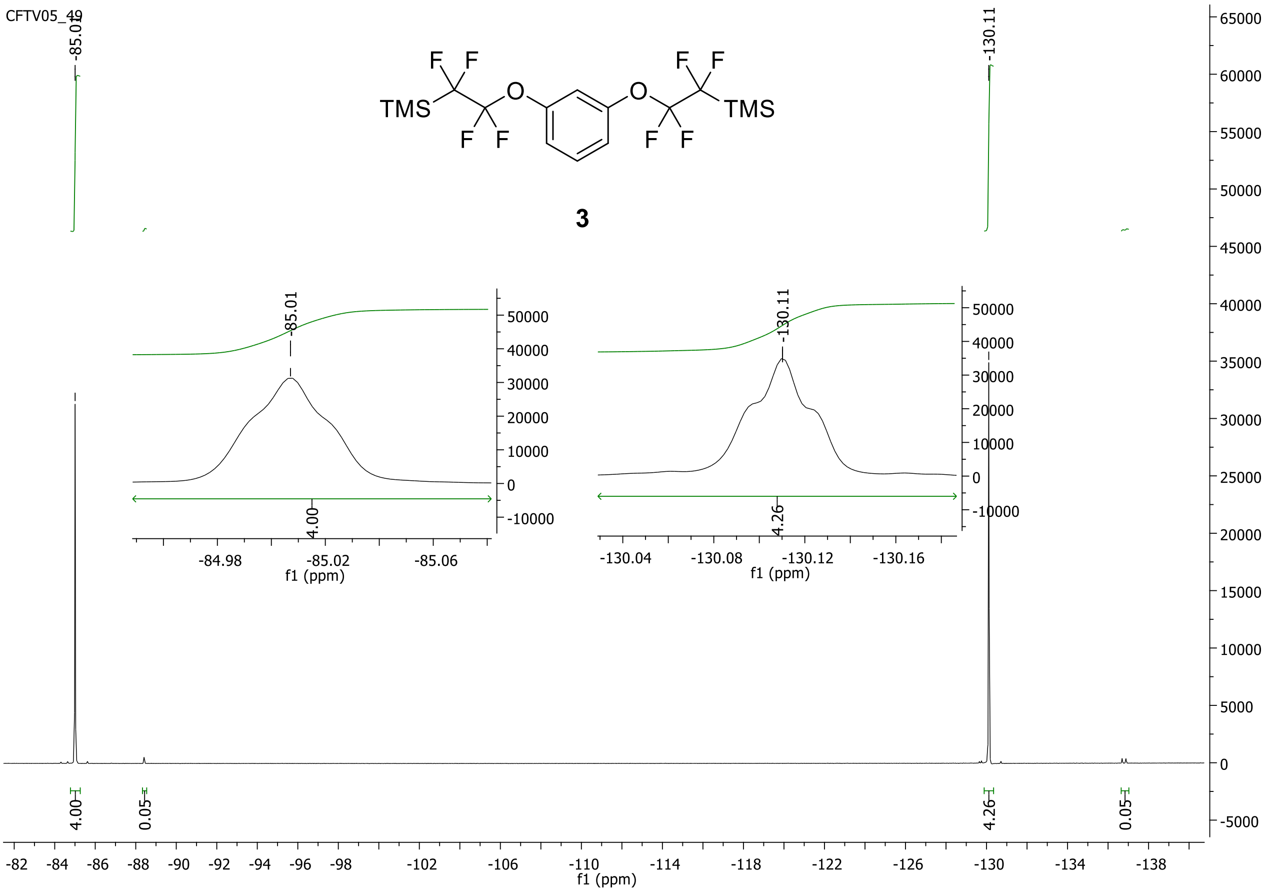


**Figure S11:** $^{19}$F NMR spectrum of bis-fluoroalkyl silane (compound **3-meta**)

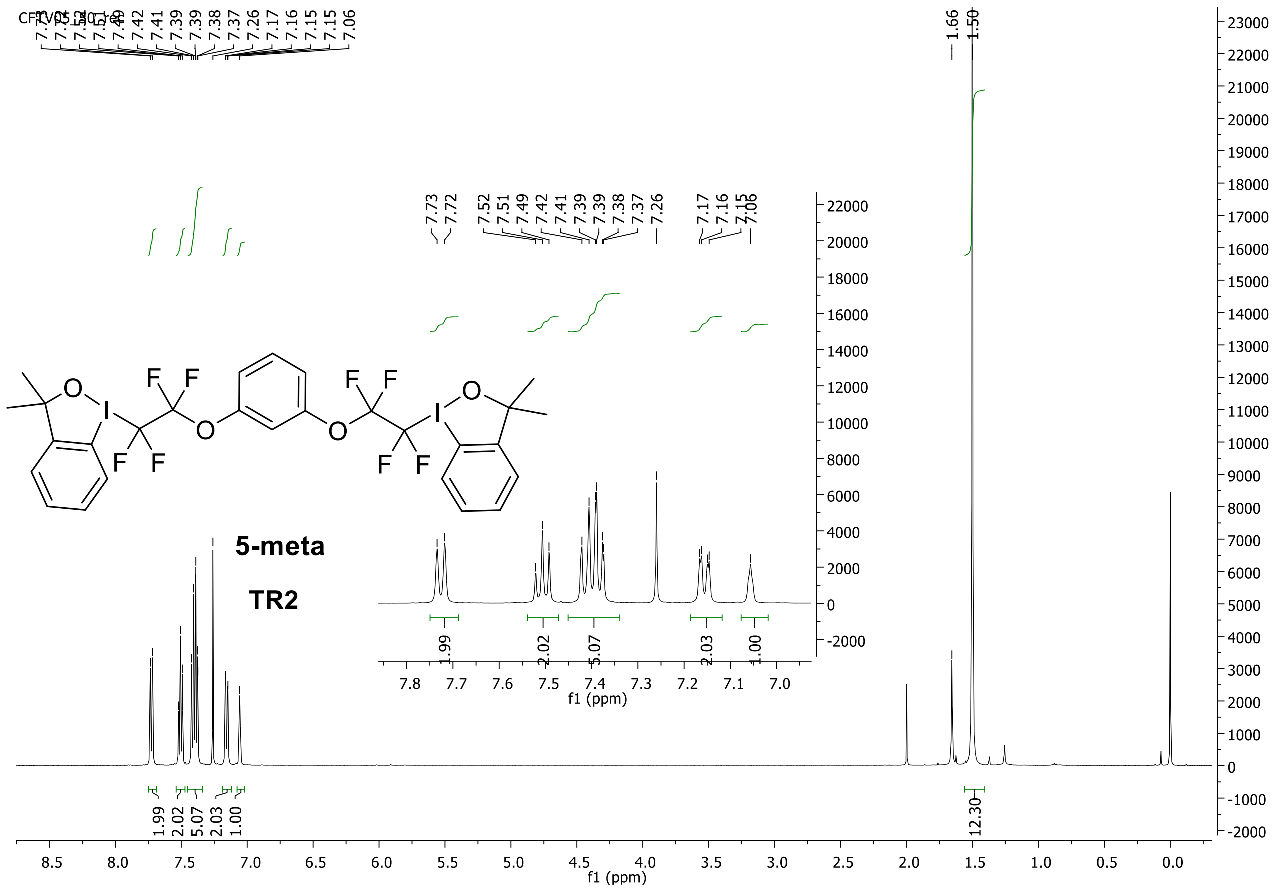

**Figure S12:** $^{1}$H NMR spectrum of Togni cross-linking agent **TR2** (compound **5-meta**)

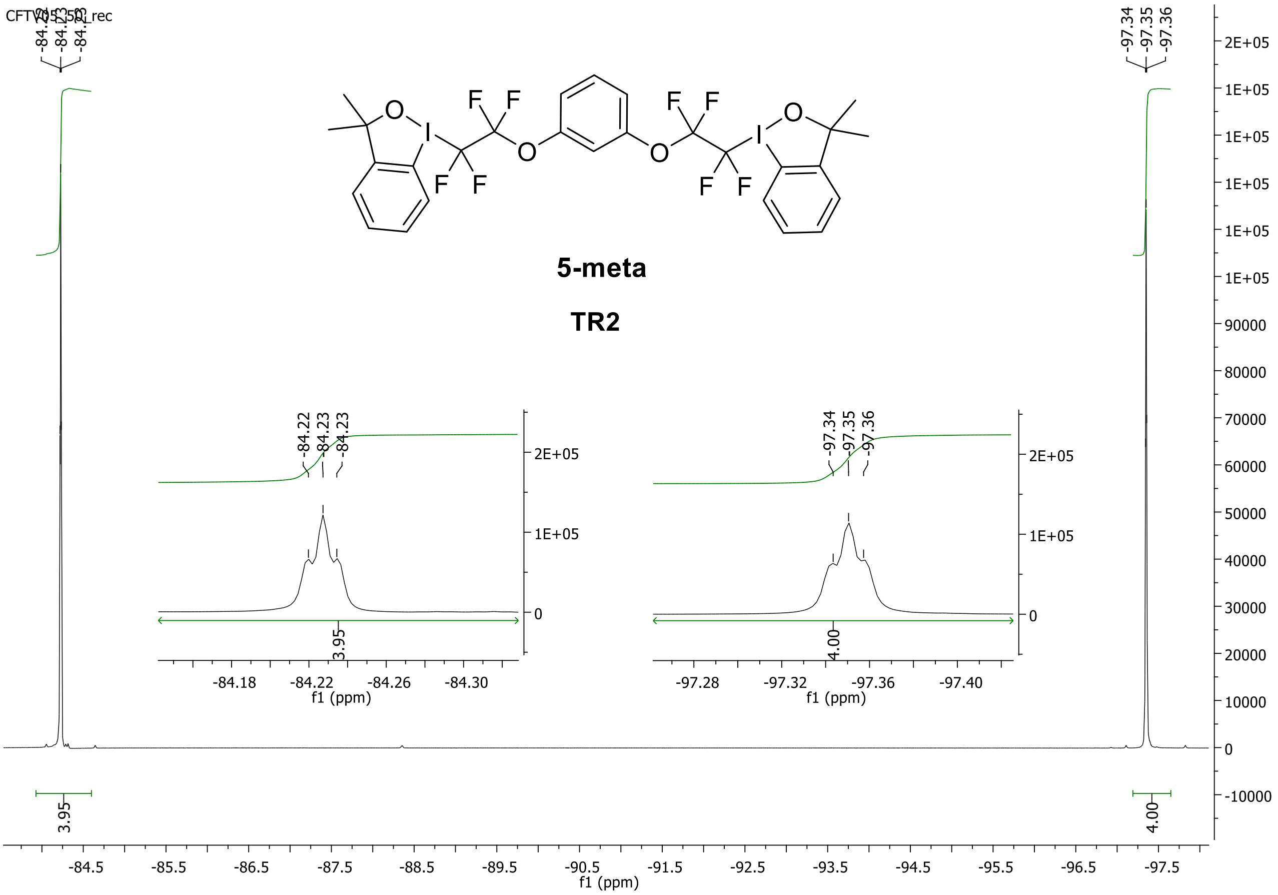


**Figure S13:** $^{19}F$ NMR spectrum of Togni cross-linking agent **TR2** (compound **5-meta**)

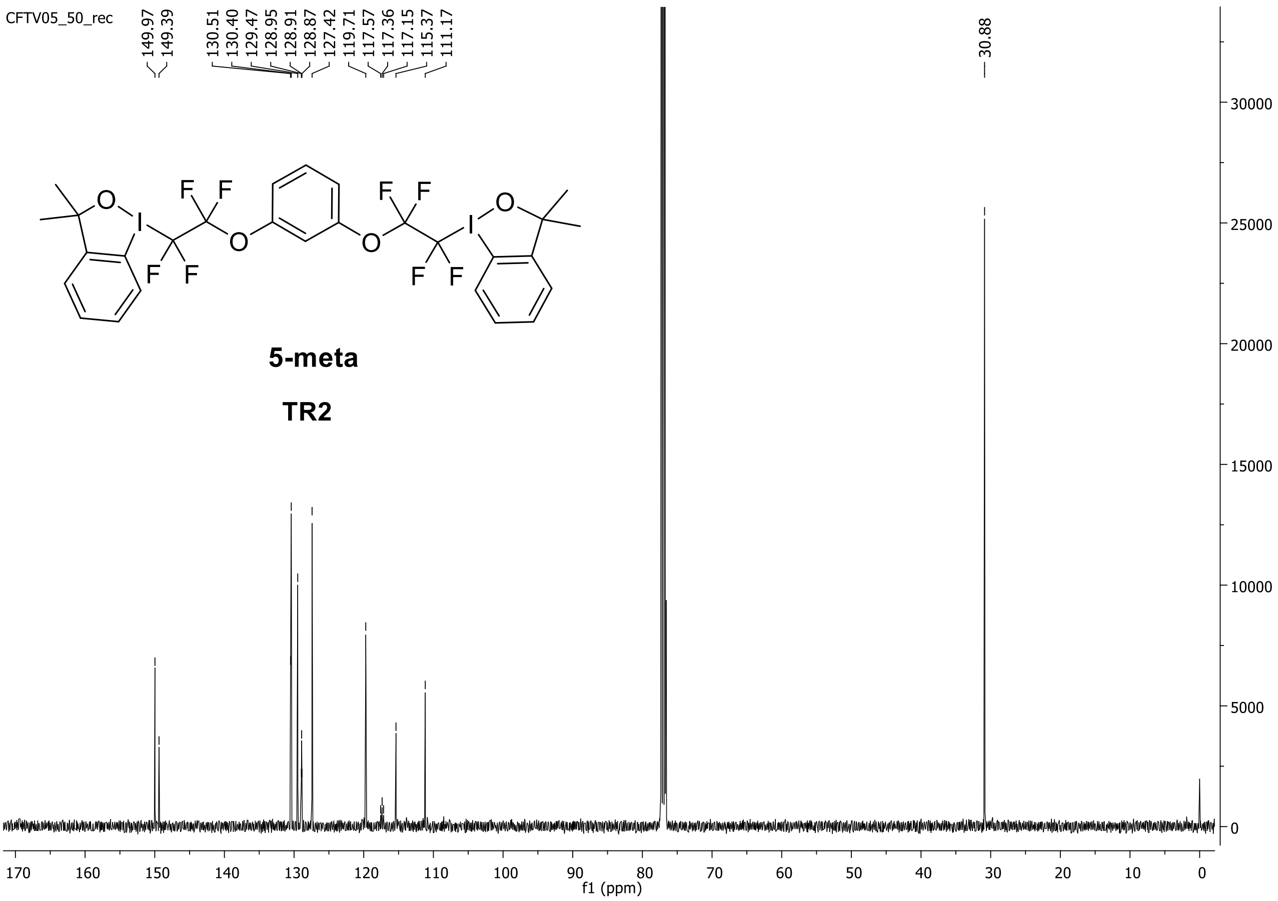


**Figure S14:** $^{13}$C NMR spectrum of Togni cross-linking agent **TR2** (compound **5-meta**)

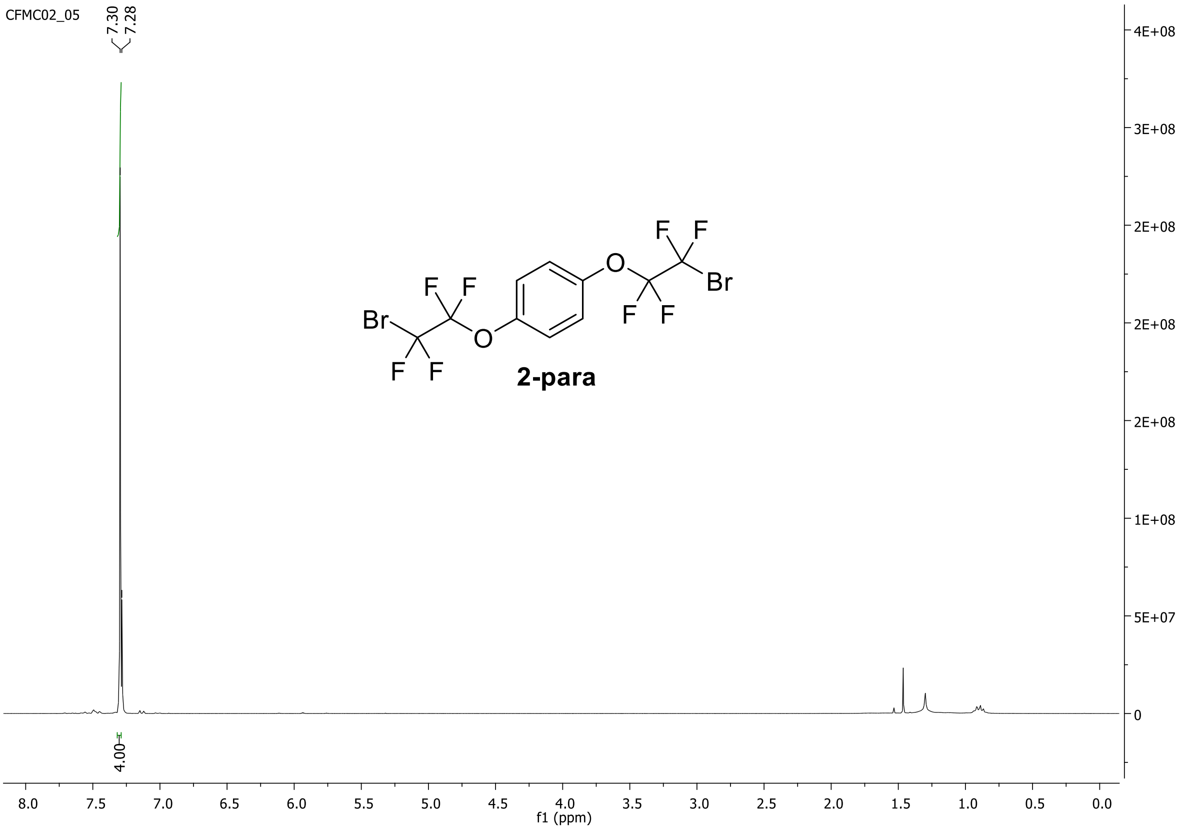


**Figure S15:** $^1$H NMR spectrum of bis-fluoroalkyl bromide (compound **2-para**)

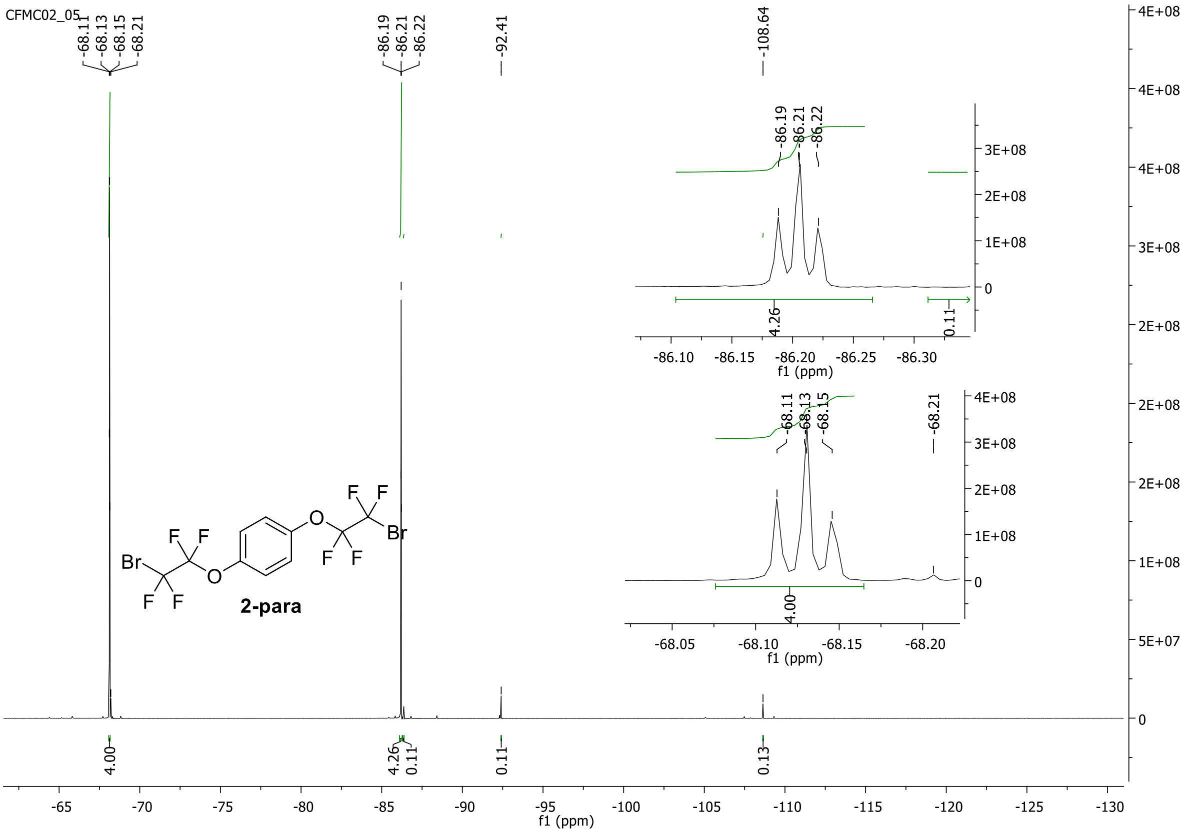


**Figure S16:** $^{19}F$ NMR spectrum of bis-fluoroalkyl bromide (compound **2-para**)

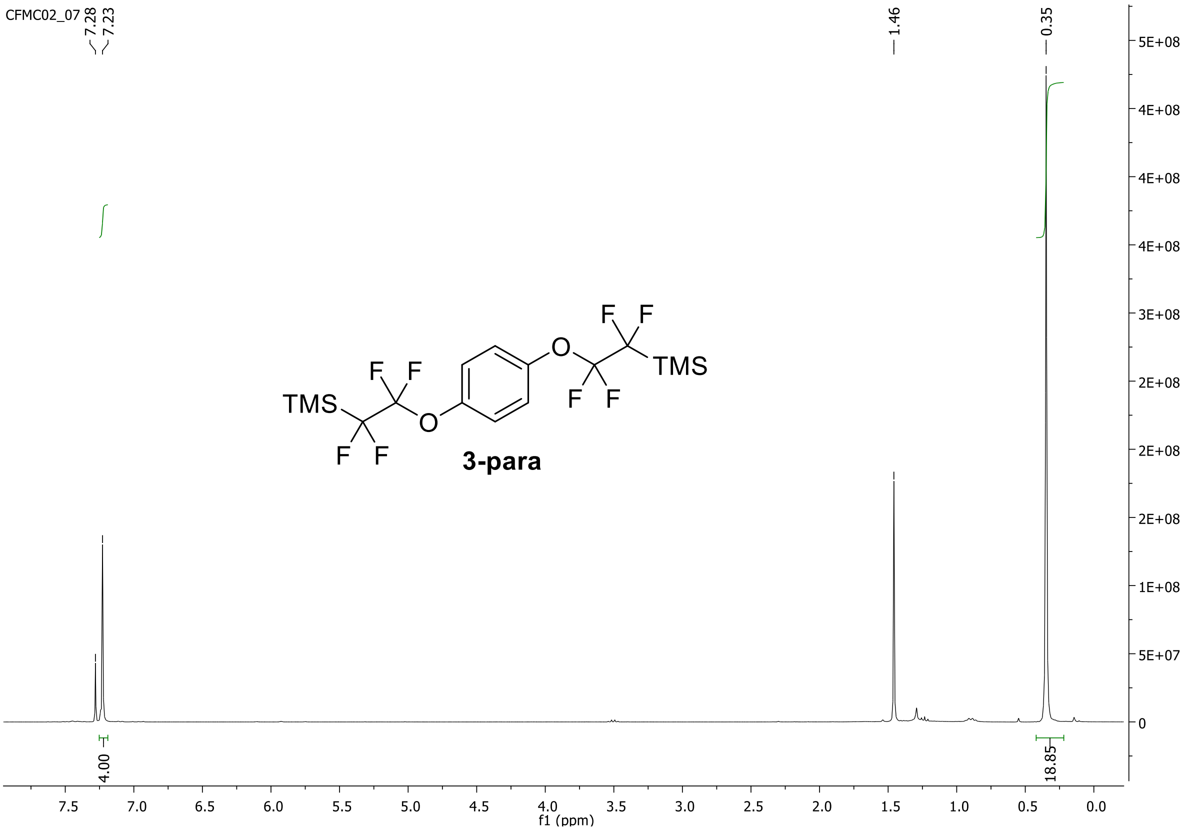


**Figure S17:** $^{1}H$ NMR spectrum of bis-fluoroalkyl silane (compound **3-para)**

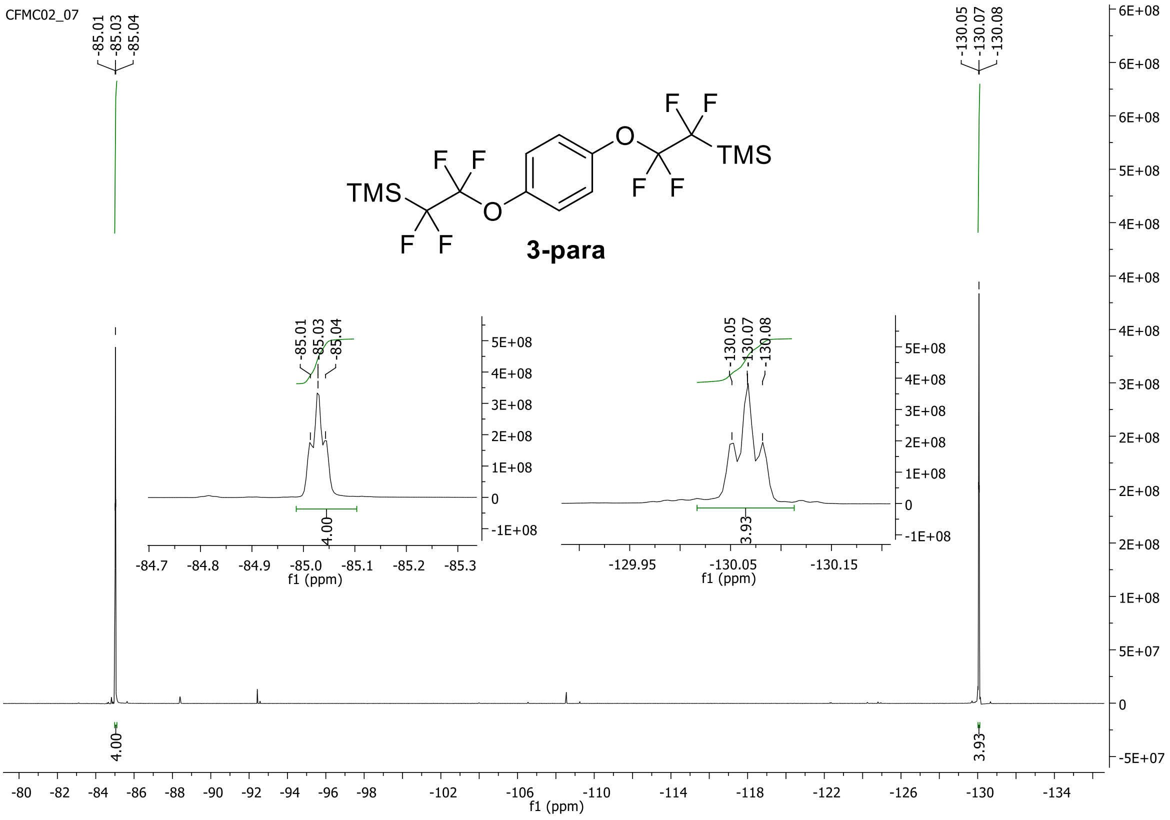


**Figure S18:** $^{19}$F NMR spectrum of bis-fluoroalkyl silane (compound **3-para**)

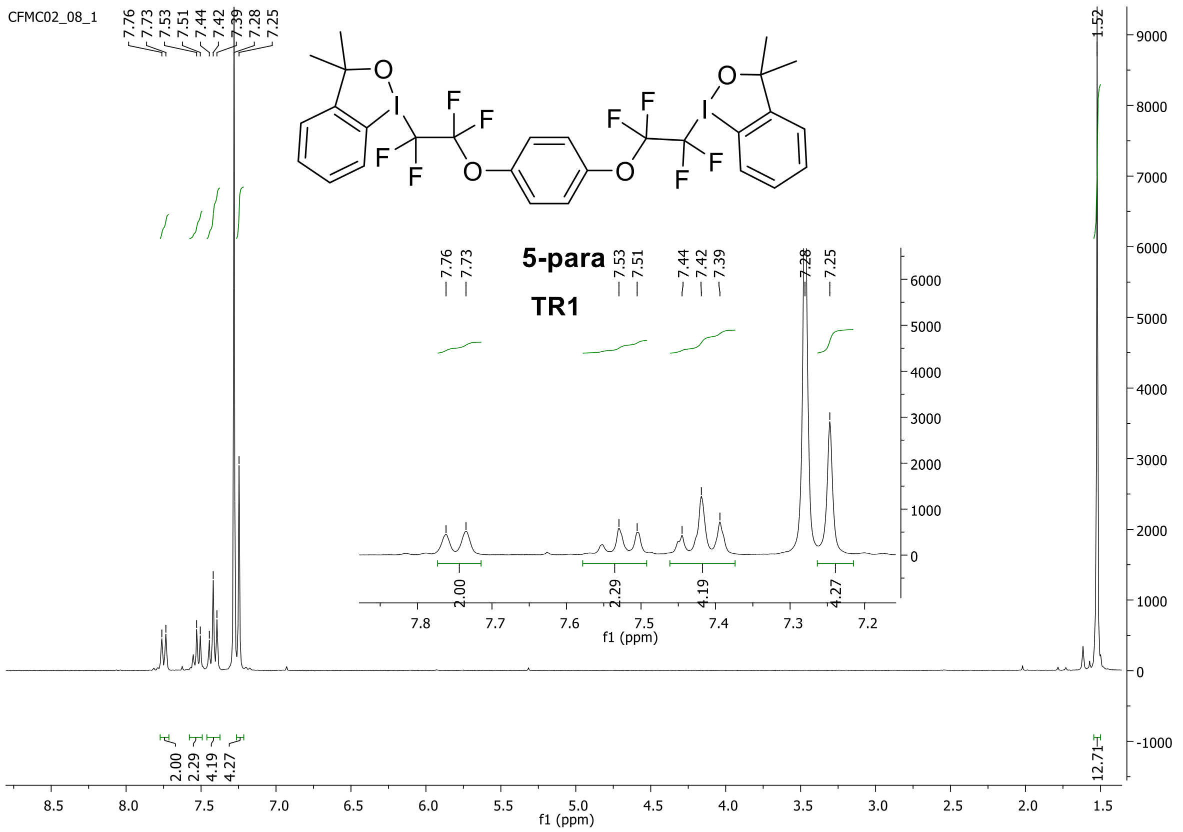


**Figure S19:** $^{1}$H NMR spectrum of Togni cross-linking agent **TR1** (compound **5-para**)

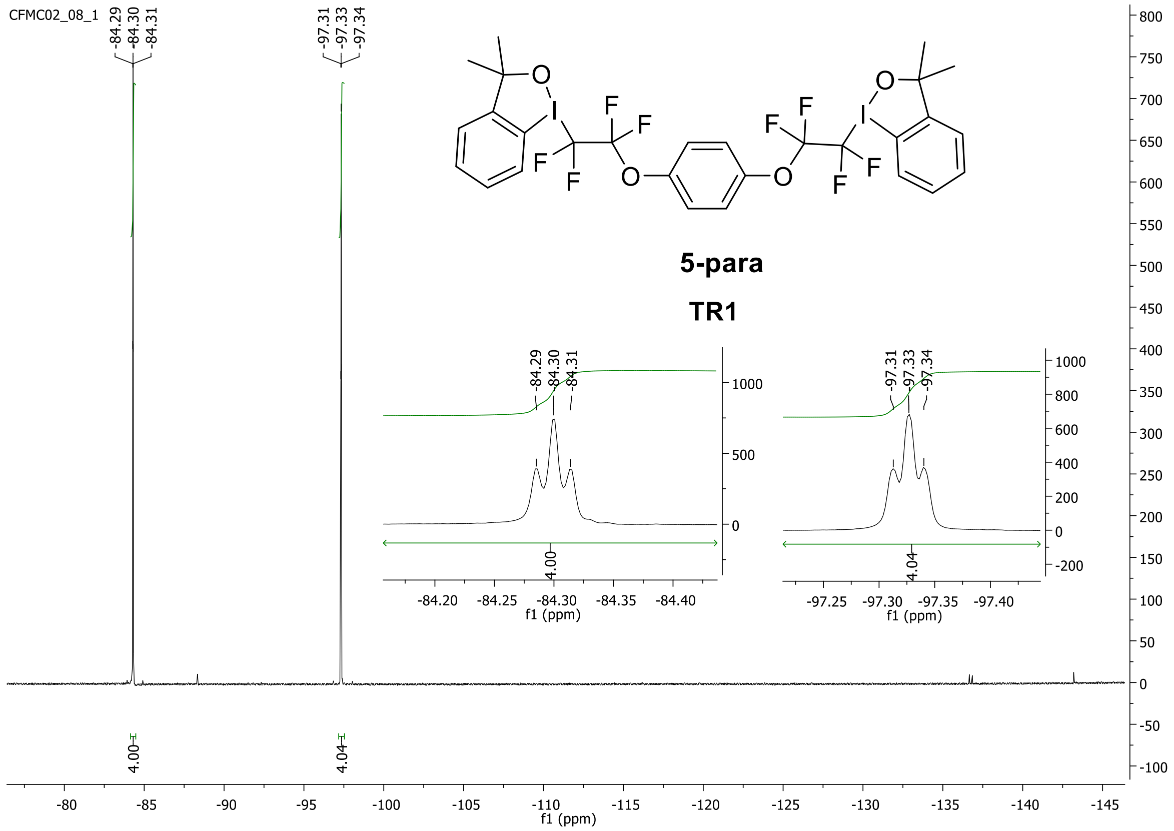


**Figure S20:** $^{19}$F NMR spectrum of Togni cross-linking agent **TR1**(compound **5-para**)

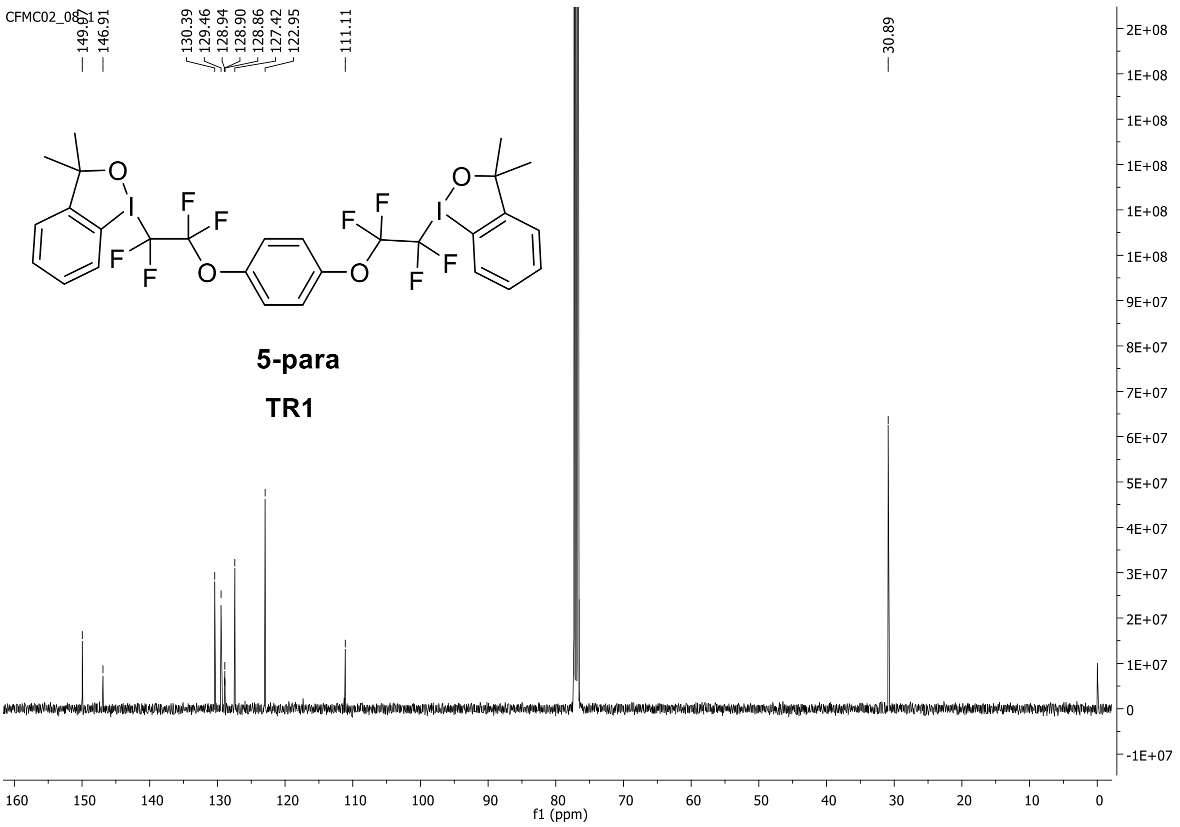


**Figure S21:** $^{13}$C NMR spectrum of Togni cross-linking agent **TR1** (compound **5-para**)

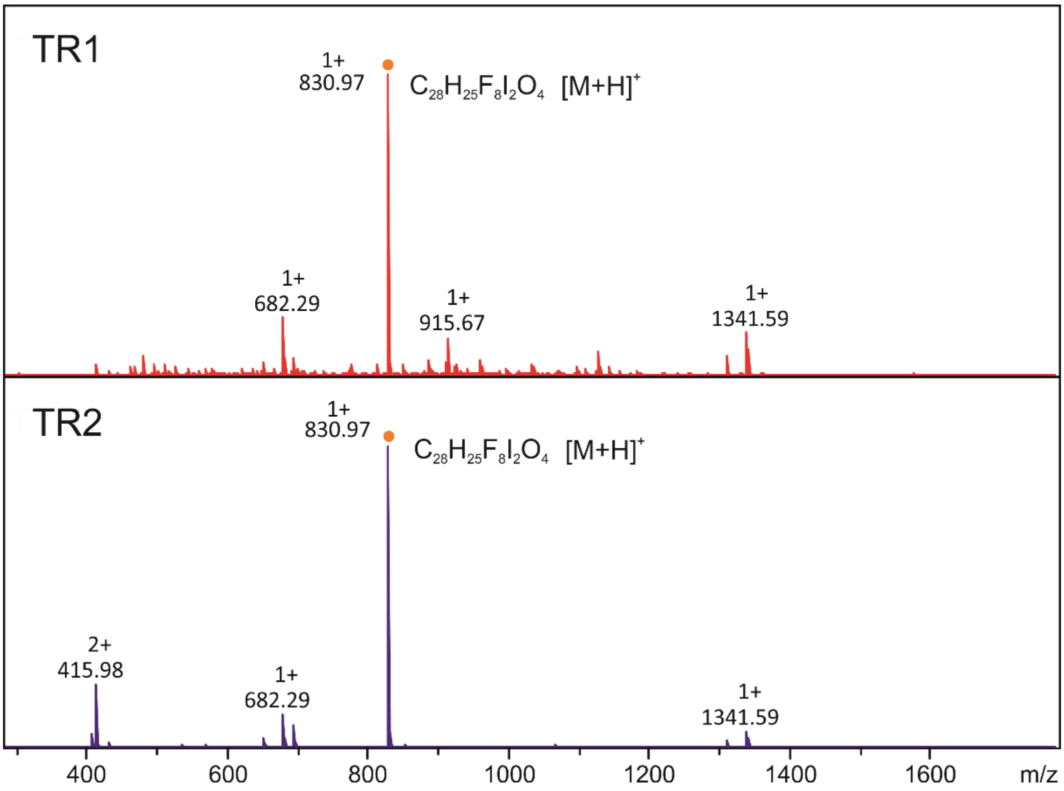


**Figure S22:** Mass spectra of Togni cross-linking reagent **TR1** (top) and Togni cross-linking reagent **TR2** (bottom). m/z 830.97 corresponds to $1^+$ charge state of compounds.

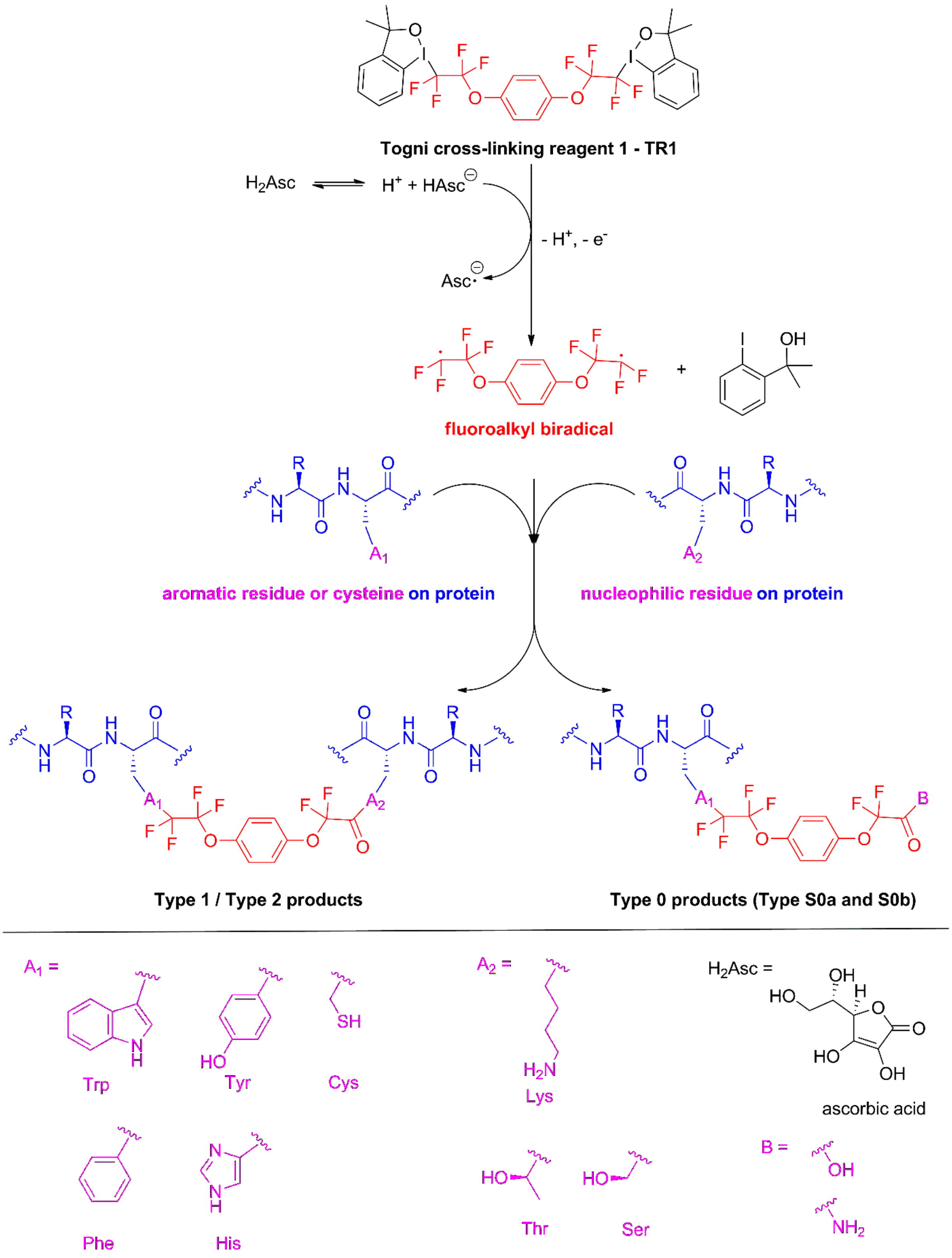


**Figure S23:** Reaction scheme of reaction steps leading to the formation of the side product generated from Togni cross-linking reagent 1 (TR1). The formation of the biradical is induced by ascorbate. One fluoroalkyl radical then undergoes stepwise degradation in water to afford the corresponding acyl fluoride that forms a covalent bond with nucleophilic residues or reacts with water molecule or ammonia molecule. Overall, the reaction steps lead to the formation of Type 1/ Type 2 products or Type 0 products with hydroxyl (Type S0a) or amine group (Type S0b).

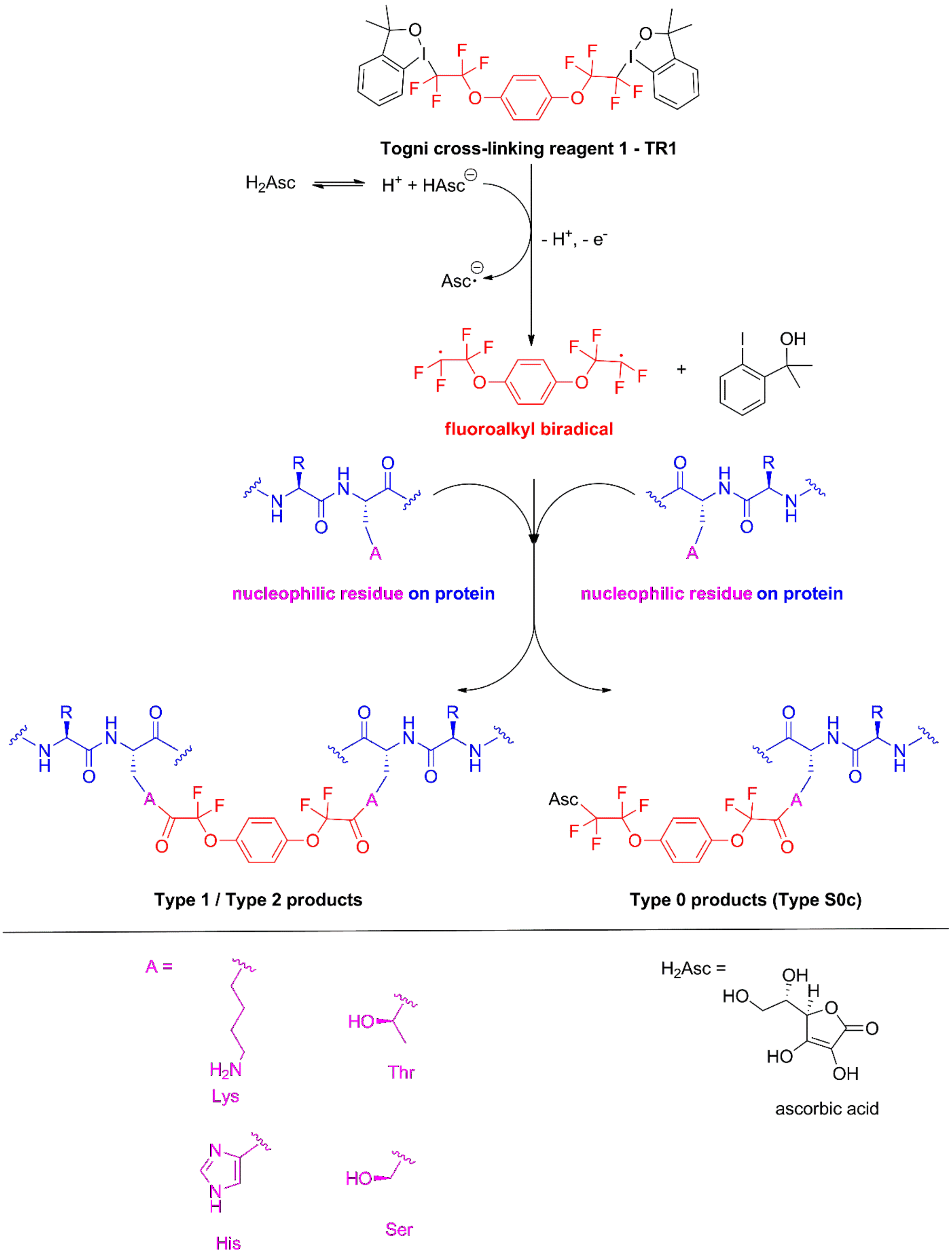


**Figure S24:** Reaction scheme of the reaction steps leading to the formation of the side product generated from Togni cross-linking reagent 1 (TR1). The formation of the biradical is induced by ascorbate. One or both fluoroalkyl radical then undergoes stepwise degradation in water to afford the corresponding acyl fluoride that forms a covalent bond with nucleophilic residues or reacts with ascorbate molecule. Overall, the reaction steps lead to the formation of Type 1/ Type 2 products or Type 0 products with ascorbate group (Type S0c).

.

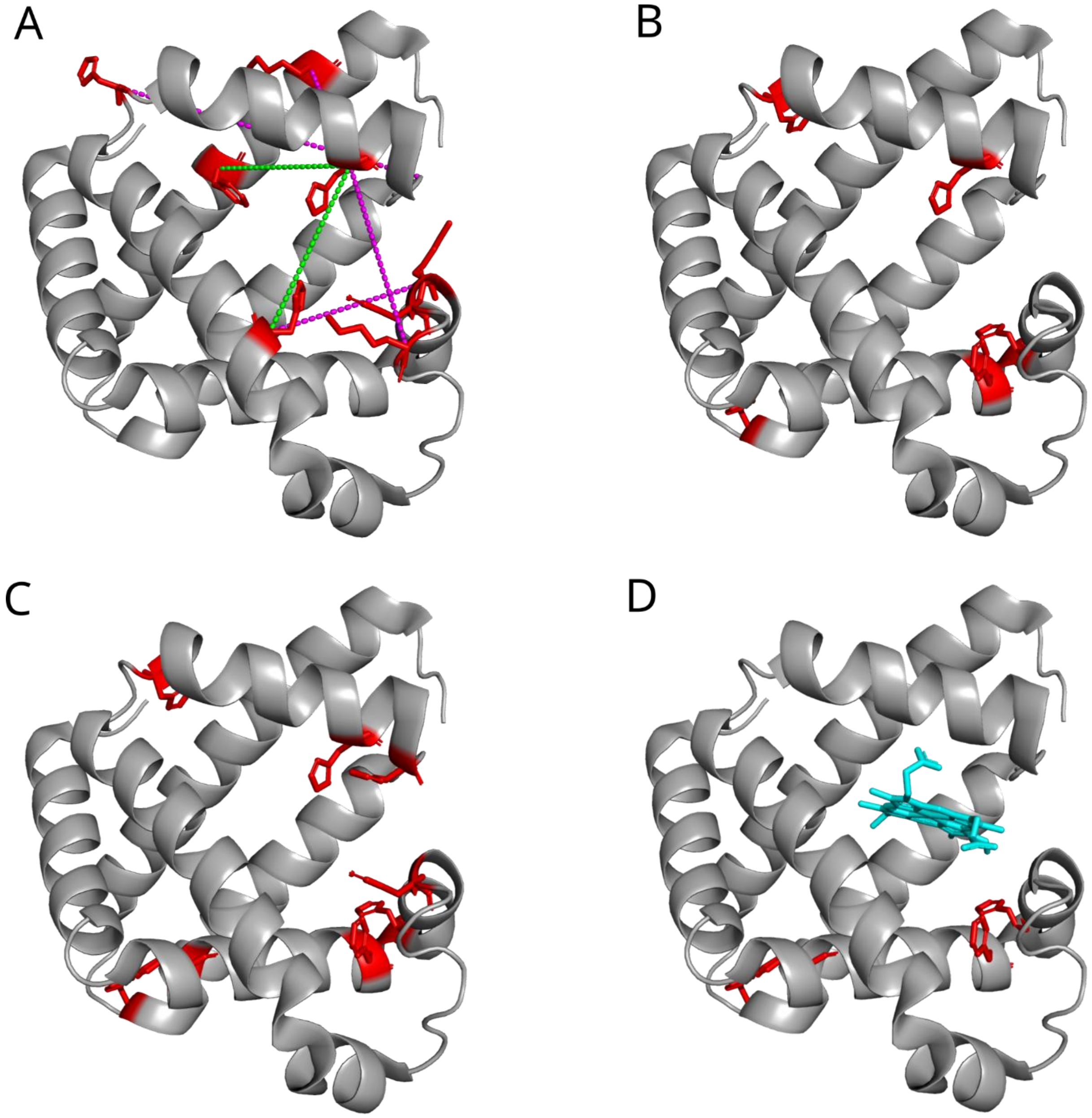


**Figure S25:** Crystal structure of horse heart apoMYO (1WLA) without heme with highlighted Type 2 products using **TR1**, main reaction products indicated in green and side products indicated in magenta (A), with highlighted residues (red) modified with Type 0 products using **TR1** (B) or **TR2** (C) and crystal structure of holoMYO with heme (cyan) and residues modified with Type 0 products highlighted in red.

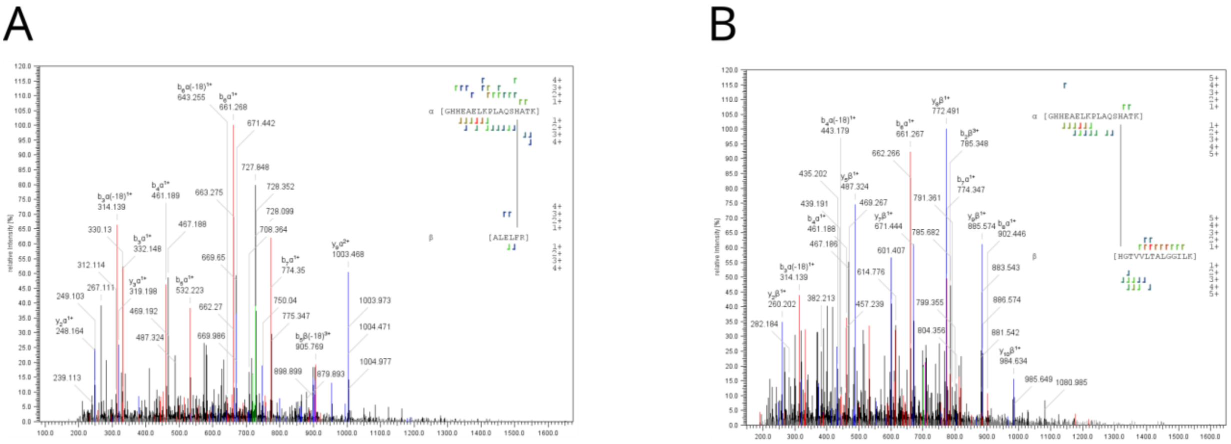


**Figure S26:** Examples of identified Type 2 products of apoMYO upon reaction with Togni cross-linking reagent **TR1** by MeroX software, specifically F138-H93(A) and H64-H93(B).

**Table S1:** List of identified main products of cross-linking reaction of apoMYO with **TR1**.

| Togni cross-linking reagent | Type of product | Exp. Mass | Theor. Mass | Deviation in ppm | Sequence | Modified peptides | Modified residues |
|---|---|---|---|---|---|---|---|
| TR1 | Type 2 | 3537.783 | 3537.789 | -1.63 | [HGTVVLTALGGILK]-[GHHEAELKPLAQSHATK] | [64-77]-[80-96] | H64-H93 |
| TR1 | Type 2 | 2907.380 | 2907.382 | -0.77 | [GHHEAELKPLAQSHATK]-[ALELFR] | [80-96]-[134-139] | H93-F138 |
| TR1 | Type 0 | 1753.683 | 1753.688 | -2.52 | [LFTGHPETLEK] | [32-42] | F33 |
| TR1 | Type 0 | 1753.684 | 1753.688 | -2.28 | [LFTGHPETLEK] | [32-42] | H36 |
| TR1 | Type 0 | 2335.978 | 2335.986 | -3.64 | [GHHEAELKPLAQSHATK] | [80-96] | H82 |
| TR1 | Type 0 | 2335.982 | 2335.986 | -1.88 | [GHHEAELKPLAQSHATK] | [80-96] | H93 |
| TR1 | Type 0 | 1984.687 | 1984.694 | -3.67 | [HPGDFGADAQGAMTK] | [119-133] | H119 |

**Table S2:** List of identified main products of cross-linking reaction of apoMYO with **TR2**.

| Togni cross-linking reagent | Type of product | Exp. Mass | Theor. Mass | Deviation in ppm | Sequence | Modified peptides | Modified residues |
|---|---|---|---|---|---|---|---|
| TR2 | Type 2 | 3345.692 | 3345.680 | 3.32 | [LFTGHPETLEKFDK]-[HGTVVLTALGGILK] | [32-45]-[64-77] | F43-H64 |
| TR2 | Type 2 | 2843.316 | 2843.319 | -0.95 | [FDKFK]-[GHHEAELKPLAQSHATK] | [43-47]-[80-96] | F43-H93 |
| TR2 | Type 2 | 2907.383 | 2907.382 | 0.23 | [GHHEAELKPLAQSHATK]-[ALELFR] | [80-96]-[134-139] | H93-F138 |
| TR2 | Type 2 | 3537.792 | 3537.789 | 0.85 | [HGTVVLTALGGILK]-[GHHEAELKPLAQSHATK] | [64-77]-[80-96] | H64-H93 |
| TR2 | Type 0 | 2088.878 | 2088.880 | -0.83 | [VEADIAGHGQEVLIR] | [17-31] | H24 |
| TR2 | Type 0 | 1753.685 | 1753.688 | -1.39 | [LFTGHPETLEK] | [32-42] | F33 |
| TR2 | Type 0 | 1753.688 | 1753.688 | -0.08 | [LFTGHPETLEK] | [32-42] | H36 |
| TR2 | Type 0 | 2143.864 | 2143.878 | -6.39 | [LFTGHPETLEKFDK] | [32-45] | F43 |
| TR2 | Type 0 | 2335.976 | 2335.986 | -4.58 | [GHHEAELKPLAQSHATK] | [80-96] | H82 |
| TR2 | Type 0 | 2335.985 | 2335.986 | -0.79 | [GHHEAELKPLAQSHATK] | [80-96] | H93 |
| TR2 | Type 0 | 1217.513 | 1217.512 | 0.56 | [HKIPIK] | [97-102] | H97 |
| TR2 | Type 0 | 1984.693 | 1984.694 | -0.44 | [HPGDFGADAQGAMTK] | [119-133] | H119 |

**Table S3:** List of identified side products of cross-linking reaction of apoMYO with **TR1**.

| Togni cross-linking reagent | Type of product | Exp. Mass | Theor. Mass | Deviation in ppm | Sequence | Modified peptides | Modified residues |
|---|---|---|---|---|---|---|---|
| TR1 | Type 2 | 3323.698 | 3323.679 | 5.76 | [LFTGHPETLEKFDK]-[HGTVVLTALGGILK] | [32-45]-[64-77] | K42-H64 |
| TR1 | Type 2 | 2821.309 | 2821.317 | -2.77 | [FDKFK]-[GHHEAELKPLAQSHATK] | [43-47]-[80-96] | K45-H93 |
| TR1 | Type 2 | 2872.438 | 2872.433 | 1.74 | [GHHEAELKPLAQSHATK]- [HKIPIK] | [80-96]-[97-102] | H81-K98 |
| TR1 | Type 2 | 3059.430 | 3059.444 | -4.62 | [GHHEAELKPLAQSHATK]-[NDIAAKYK] | [80-96]-[140-147] | H93-K145 |
| TR1 | Type 1 | 1945.838 | 1945.844 | -3.06 | [LFTGHPETLEKFDK] | [32-45] | K42-F43 |
| TR1 | Type 1 | 1752.930 | 1752.930 | -0.56 | [KHGTVVLTALGGILK] | [63-77] | K63-T66 |
| TR1 | Type 1 | 2099.953 | 2099.956 | -1.13 | [GHHEAELKPLAQSHATK] | [80-96] | S92-T95 |
| TR1 | Type 1 | 2137.951 | 2137.952 | -0.84 | [GHHEAELKPLAQSHATK] | [80-96] | H82-K87 |
| TR1 | Type 1 | 2403.107 | 2403.106 | 0.26 | [GHHEAELKPLAQSHATKHK] | [80-98] | K96-H97 |

**Table S4:** List of identified side products of cross-linking reaction of apoMYO with **TR2**.

| Togni cross-linking reagent | Type of product | Exp. Mass | Theor. Mass | Deviation in ppm | Sequence | Modified peptides | Modified residues |
|---|---|---|---|---|---|---|---|
| TR2 | Type 2 | 4063.948 | 4063.952 | -0.99 | [LFTGHPETLEKFDK]-[GHHEAELKPLAQSHATKHK] | [32-45]-[80-98] | F43-K96 |
| TR2 | Type 2 | 3760.779 | 3760.802 | -6.87 | [LFTGHPETLEKFDK]- [GHHEAELKPLAQSHATK] | [32-45] -[80-96] | T39-S92 |
| TR2 | Type 2 | 3798.795 | 3798.799 | -1.04 | [LFTGHPETLEKFDK]-[GHHEAELKPLAQSHATK] | [32-45]-[80-96] | K42-H93 |
| TR2 | Type 2 | 2821.314 | 2821.317 | -0.88 | [FDKFK]-[GHHEAELKPLAQSHATK] | [43-47]-[80-96] | K45-H93 |
| TR2 | Type 2 | 3780.954 | 3780.941 | 3.47 | [HGTVVLTALGGILK]-[GHHEAELKPLAQSHATKHK] | [64-77]-[80-98] | H64-K96 |
| TR2 | Type 2 | 3515.805 | 3515.787 | 5.12 | [HGTVVLTALGGILK]- [GHHEAELKPLAQSHATK] | [64-77]-[80-96] | T66-H93 |
| TR2 | Type 2 | 2872.422 | 2872.433 | -3.83 | [GHHEAELKPLAQSHATK]- [HKIPIK] | [80-96]-[97-102] | H93-K98 |
| TR2 | Type 2 | 2885.372 | 2885.380 | -2.92 | [GHHEAELKPLAQSHATK]-[ALELFR] | [80-96]-[134-139] | K87-F138 |
| TR2 | Type 2 | 3059.435 | 3059.444 | -3.17 | [GHHEAELKPLAQSHATK]-[NDIAAKYK] | [80-96]-[140-147] | H82-K145 |
| TR2 | Type 1 | 2221.011 | 2221.008 | 1.44 | [LFTGHPETLEKFDKFK] | [32-47] | K42-F43 |
| TR2 | Type 1 | 2137.950 | 2137.952 | -1.16 | [GHHEAELKPLAQSHATK] | [80-96] | H82-K87 |
| TR2 | Type 1 | 2403.102 | 2403.106 | -1.98 | [GHHEAELKPLAQSHATKHK] | [80-98] | H93-K96 |

**Table S5:** List of identified Type 0 side products of cross-linking reaction of apoMYO with **TR1**.

| Togni cross-linking reagent | Type of product | Exp. Mass | Theor. Mass | Deviation in ppm | Sequence | Modified peptides | Modified residues |
|---|---|---|---|---|---|---|---|
| TR1 | Type S0a | 1573.660 | 1573.664 | 2.49 | [LFTGHPETLEK] | [32-42] | F33 |
| TR1 | Type S0a | 1573.661 | 1573.664 | 2.26 | [LFTGHPETLEK] | [32-42] | H36 |
| TR1 | Type S0a | 1963.853 | 1963.855 | 0.83 | [LFTGHPETLEKFDK] | [32-45] | F43 |
| TR1 | Type S0a | 2155.958 | 2155.963 | 2.29 | [GHHEAELKPLAQSHATK] | [80-96] | H93 |
| TR1 | Type S0a | 2155.954 | 2155.963 | 3.91 | [GHHEAELKPLAQSHATK] | [80-96] | H82 |
| TR1 | Type S0a | 2421.119 | 2421.117 | -0.74 | [GHHEAELKPLAQSHATKHK] | [80-98] | H93 |
| TR1 | Type S0a | 1820.662 | 1820.666 | 2.21 | [HPGDFGADAQGAMTK] + 1 oxidation | [119-133] | H119 |
| TR1 | Type S0a | 1820.664 | 1820.666 | 1.01 | [HPGDFGADAQGAMTK] + 1 oxidation | [119-133] | F123 |
| TR1 | Type S0a | 1050.435 | 1050.437 | 1.36 | [ALELFR] | [134-139] | F138 |
| TR1 | Type S0b | 1572.681 | 1572.680 | -0.12 | [LFTGHPETLEK] | [32-42] | F33 |
| TR1 | Type S0b | 1679.858 | 1679.859 | 0.72 | [HGTVVLTALGGILK] | [64-77] | H64 |
| TR1 | Type S0b | 2154.978 | 2154.979 | 0.51 | [GHHEAELKPLAQSHATK] | [80-96] | H82 |
| TR1 | Type S0b | 2154.950 | 2154.979 | 13.43 | [GHHEAELKPLAQSHATK] | [80-96] | H93 |
| TR1 | Type S0b | 1803.686 | 1803.687 | 0.57 | [HPGDFGADAQGAMTK] | [119-133] | H119 |
| TR1 | Type S0c | 2121.862 | 2121.876 | 6.68 | [LFTGHPETLEKFDK] | [32-45] | K42 |
| TR1 | Type S0c | 1966.953 | 1966.960 | 3.23 | [HGTVVLTALGGILKK] | [64-78] | K77 |
| TR1 | Type S0c | 2313.975 | 2313.985 | 3.94 | [GHHEAELKPLAQSHATK] | [80-96] | H82 |
| TR1 | Type S0c | 2313.977 | 2313.985 | 3.11 | [GHHEAELKPLAQSHATK] | [80-96] | K87 |
| TR1 | Type S0c | 2313.985 | 2313.985 | -0.39 | [GHHEAELKPLAQSHATK] | [80-96] | H93 |
| TR1 | Type S0c | 2579.125 | 2579.138 | 4.94 | [GHHEAELKPLAQSHATKHK] | [80-98] | K96 |
| TR1 | Type S0c | 1195.510 | 1195.510 | 0.64 | [HKIPIK] | [97-102] | K98 |

**Table S6:** List of identified Type 0 side products of cross-linking reaction of apoMYO with **TR2**.

| Togni cross-linking reagent | Type of product | Exp. Mass | Theor. Mass | Deviation in ppm | Sequence | Modified peptides | Modified residues |
|---|---|---|---|---|---|---|---|
| TR2 | Type S0a | 1908.854 | 1908.856 | 1.10 | [VEADIAGHGQEVLIR] | [17-31] | H24 |
| TR2 | Type S0a | 1573.658 | 1573.664 | 3.77 | [LFTGHPETLEK] | [32-42] | H36 |
| TR2 | Type S0a | 1573.664 | 1573.664 | 0.07 | [LFTGHPETLEK] | [32-42] | F33 |
| TR2 | Type S0a | 1963.851 | 1963.855 | 1.64 | [LFTGHPETLEKFDK] | [32-45] | F43 |
| TR2 | Type S0a | 1680.850 | 1680.843 | -4.41 | [HGTVVLTALGGILK] | [64-77] | H64 |
| TR2 | Type S0a | 2155.955 | 2155.963 | 3.51 | [GHHEAELKPLAQSHATK] | [80-96] | H93 |
| TR2 | Type S0a | 2155.957 | 2155.963 | 2.66 | [GHHEAELKPLAQSHATK] | [80-96] | H82 |
| TR2 | Type S0a | 1037.490 | 1037.490 | -0.76 | [HKIPIK] | [97-102] | H97 |
| TR2 | Type S0a | 1804.663 | 1804.671 | 4.17 | [HPGDFGADAQGAMTK] | [119-133] | H119 |
| TR2 | Type S0b | 1907.871 | 1907.872 | 0.81 | [VEADIAGHGQEVLIR] | [17-31] | H24 |
| TR2 | Type S0b | 1572.683 | 1572.680 | -1.72 | [LFTGHPETLEK] | [32-42] | F33 |
| TR2 | Type S0b | 1572.678 | 1572.680 | 1.81 | [LFTGHPETLEK] | [32-42] | H36 |
| TR2 | Type S0b | 1962.869 | 1962.871 | 0.63 | [LFTGHPETLEKFDK] | [32-45] | F43 |
| TR2 | Type S0b | 2154.982 | 2154.980 | -1.15 | [GHHEAELKPLAQSHATK] | [80-96] | H93 |
| TR2 | Type S0b | 1803.685 | 1803.687 | 0.86 | [HPGDFGADAQGAMTK] | [119-133] | H119 |
| TR2 | Type S0c | 2121.872 | 2121.876 | 1.78 | [LFTGHPETLEKFDK] | [32-45] | K42 |
| TR2 | Type S0c | 1939.752 | 1939.759 | 3.61 | [TEAEMKASEDLKK] | [51-63] | K56 |
| TR2 | Type S0c | 2442.080 | 2442.080 | -0.19 | [KGHHEAELKPLAQSHATK] | [79-96] | K79 |
| TR2 | Type S0c | 2313.983 | 2313.985 | 0.65 | [GHHEAELKPLAQSHATK] | [80-96] | H93 |
| TR2 | Type S0c | 2313.981 | 2313.985 | 1.40 | [GHHEAELKPLAQSHATK] | [80-96] | K87 |
| TR2 | Type S0c | 2313.978 | 2313.985 | 2.76 | [GHHEAELKPLAQSHATK] | [80-96] | H82 |
| TR2 | Type S0c | 2579.145 | 2579.138 | -2.69 | [GHHEAELKPLAQSHATKHK] | [80-98] | K96 |
| TR2 | Type S0c | 1195.513 | 1195.510 | -1.91 | [HKIPIK] | [97-102] | K98 |

**Table S7:** List of identified main products of cross-linking reaction of holoMYO with **TR1**.

| Togni cross-linking reagent | Type of product | Exp. Mass | Theor. Mass | Deviation in ppm | Sequence | Modified peptides | Modified residues |
|---|---|---|---|---|---|---|---|
| TR1 | Type 0 | 2088.879 | 2088.880 | -0.12 | [VEADIAGHGQEVLIR] | [17-31] | H24 |
| TR1 | Type 0 | 1753.696 | 1753.688 | 4.91 | [LFTGHPETLEK] | [32-42] | F33 |
| TR1 | Type 0 | 1753.693 | 1753.688 | 2.94 | [LFTGHPETLEK] | [32-42] | H36 |
| TR1 | Type 0 | 1984.701 | 1984.694 | 3.39 | [HPGDFGADAQGAMTK] | [119-133] | H119 |

**Table S8:** List of identified main products of cross-linking reaction of holoMYO with **TR2**.

| Togni cross-linking reagent | Type of product | Exp. Mass | Theor. Mass | Deviation in ppm | Sequence | Modified peptides | Modified residues |
|---|---|---|---|---|---|---|---|
| TR2 | Type 0 | 2088.887 | 2088.880 | 3.61 | [VEADIAGHGQEVLIR] | [17-31] | H24 |
| TR2 | Type 0 | 1753.690 | 1753.688 | 1.24 | [LFTGHPETLEK] | [32-42] | F33 |
| TR2 | Type 0 | 2143.872 | 2143.878 | -2.83 | [LFTGHPETLEKFDK] | [32-45] | F43 |
| TR2 | Type 0 | 1984.698 | 1984.694 | 2.24 | [HPGDFGADAQGAMTK] | [119-133] | H119 |

**Table S9:** List of identified side products of cross-linking reaction of holoMYO with **TR1**.

| Togni cross-linking reagent | Type of product | Exp. Mass | Theor. Mass | Deviation in ppm | Sequence | Modified peptides | Modified residues |
|---|---|---|---|---|---|---|---|
| TR1 | Type 1 | 1945.845 | 1945.844 | 0.60 | [LFTGHPETLEKFDK] | [32-45] | K42-F43 |

**Table S10:** List of identified side products of cross-linking reaction of holoMYO with **TR2**.

| Togni cross-linking reagent | Type of product | Exp. Mass | Theor. Mass | Deviation in ppm | Sequence | Modified peptides | Modified residues |
|---|---|---|---|---|---|---|---|
| TR2 | Type 2 | 3605.844 | 3605.823 | 5.99 | [GLSDGEWQQVLNVWGK]-[HGTVVLTALGGILKK] | [1-16]- [64-78] | W14-K77 |
| TR2 | Type 1 | 1945.844 | 1945.844 | -0.34 | [LFTGHPETLEKFDK] | [32-45] | K42-F43 |
| TR2 | Type 1 | 2221.009 | 2221.008 | 0.44 | [LFTGHPETLEKFDKFK] | [32-47] | F43-K45 |

**Table S11:** List of identified Type 0 side products of cross-linking reaction of holoMYO with **TR1**.

| Togni cross-linking reagent | Type of product | Exp. Mass | Theor. Mass | Deviation in ppm | Sequence | Modified peptides | Modified residues |
|---|---|---|---|---|---|---|---|
| TR1 | Type S0a | 1908.860 | 1908.856 | -1.92 | [VEADIAGHGQEVLIR] | [17-31] | H24 |
| TR1 | Type S0a | 1573.672 | 1573.664 | -5.19 | [LFTGHPETLEK] | [32-42] | F33 |
| TR1 | Type S0a | 1573.668 | 1573.664 | -2.36 | [LFTGHPETLEK] | [32-42] | H36 |
| TR1 | Type S0a | 1804.676 | 1804.671 | -2.99 | [HPGDFGADAQGAMTK] | [119-133] | H119 |
| TR1 | Type S0a | 1820.672 | 1820.666 | -3.64 | [HPGDFGADAQGAMTK] + 1 oxidation | [119-133] | F123 |
| TR1 | Type S0a | 1050.435 | 1050.437 | 1.23 | [ALELFR] | [134-139] | F138 |
| TR1 | Type S0b | 1907.873 | 1907.872 | -0.44 | [VEADIAGHGQEVLIR] | [17-31] | H24 |
| TR1 | Type S0b | 1572.685 | 1572.680 | -2.72 | [LFTGHPETLEK] | [32-42] | F33 |
| TR1 | Type S0b | 1803.692 | 1803.687 | -3.07 | [HPGDFGADAQGAMTK] | [119-133] | H119 |
| TR1 | Type S0c | 2121.876 | 2121.876 | 0.07 | [LFTGHPETLEKFDK] | [32-45] | K42 |
| TR1 | Type S0c | 1562.583 | 1562.579 | -2.61 | [HLKTEAEMK] + 1 oxidation | [48-56] | K50 |
| TR1 | Type S0c | 1955.755 | 1955.754 | -0.45 | [TEAEMKASEDLKK] +1 oxidation | [51-63] | K56 |
| TR1 | Type S0c | 1966.968 | 1966.960 | -4.28 | [HGTVVLTALGGILKK] | [64-78] | K77 |
| TR1 | Type S0c | 2313.989 | 2313.985 | -1.95 | [GHHEAELKPLAQSHATK] | [80-96] | K87 |

**Table S12:** List of identified Type 0 side products of cross-linking reaction of holoMYO with **TR2**.

| Togni cross-linking reagent | Type of product | Exp. Mass | Theor. Mass | Deviation in ppm | Sequence | Modified peptides | Modified residues |
|---|---|---|---|---|---|---|---|
| TR2 | Type0B | 1573.666 | 1573.664 | -0.82 | [LFTGHPETLEK] | [32-42] | F33 |
| TR2 | Type S0a | 1573.668 | 1573.664 | -2.40 | [LFTGHPETLEK] | [32-42] | H36 |
| TR2 | Type S0a | 1804.676 | 1804.671 | -3.08 | [HPGDFGADAQGAMTK] | [119-133] | H119 |
| TR2 | Type S0a | 1050.435 | 1050.437 | 1.59 | [ALELFR] | [134-139] | F138 |
| TR2 | Type S0b | 1803.692 | 1803.687 | -2.95 | [HPGDFGADAQGAMTK] | [119-133] | H119 |
| TR2 | Type S0c | 2121.873 | 2121.876 | 1.58 | [LFTGHPETLEKFDK] | [32-45] | K42 |
| TR2 | Type S0c | 1546.587 | 1546.584 | -2.06 | [HLKTEAEMK] | [48-56] | K50 |
| TR2 | Type S0c | 1966.967 | 1966.960 | -3.93 | [HGTVVLTALGGILKK] | [64-78] | K77 |
| TR2 | Type S0c | 2313.989 | 2313.985 | -1.76 | [GHHEAELKPLAQSHATK] | [80-96] | K87 |

**Table S13:** List of identified main products of cross-linking reaction of RHOA with **TR2** using FFAP.

| Togni cross-linking reagent | Type of product | Exp. Mass | Theor. Mass | Deviation in ppm | Sequence | Modified peptides | Modified residues |
|---|---|---|---|---|---|---|---|
| TR2 | Type 2 | 2300.964 | 2300.968 | -1.62 | [IGAFGYMECSAK]-[SGCLVLK] | [156-167]-[193-199] | C164-C195 |
| TR2 | Type 0 | 1513.588 | 1513.580 | 5.23 | [LVIVGDGACGK] | [13-23] | C21 |
| TR2 | Type 0 | 2160.978 | 2160.971 | 3.30 | [HFCPNVPIILVGNKK] | [110-124] | C112 |
| TR2 | Type 0 | 1480.493 | 1480.486 | 5.05 | [EVFEMATR] | [174-181] | F176 |
| TR2 | Type 0 | 1201.437 | 1201.437 | 0.41 | [SGCLVLK] | [193-199] | C195 |

**Table S14:** List of identified main products of cross-linking reaction of RHOA with **TR2** without ascorbate activation.

| Togni cross-linking reagent | Type of product | Exp. Mass | Theor. Mass | Deviation in ppm | Sequence | Modified peptides | Modified residues |
|---|---|---|---|---|---|---|---|
| TR2 | Type 2 | 3992.874 | 3992.856 | 4.35 | [QVELALWDTAGQEDYDR]-[HFCPNVPIILVGNKK] | [57-73]-[110-124] | W63-C112 |
| TR2 | Type 2 | 3260.509 | 3260.502 | 2.24 | [HFCPNVPIILVGNKK]-[IGAFGYMECSAK] | [110-124]-[156-167] | C112-C164 |
| TR2 | Type 2 | 2703.350 | 2703.344 | 2.52 | [HFCPNVPIILVGNKK]-[SGCLVLK] | [110-124]-[193-199] | C112-C195 |
| TR2 | Type 2 | 2300.977 | 2300.968 | 4.13 | [IGAFGYMECSAK]-[SGCLVLK] | [156-167]-[193-199] | C164-C195 |

**Table S15:** List of identified side products of cross-linking reaction of RHOA with **TR2** using FFAP.

| Togni cross-linking reagent | Type of product | Exp. Mass | Theor. Mass | Deviation in ppm | Sequence | Modified peptides | Modified residues |
|---|---|---|---|---|---|---|---|
| TR2 | Type 1 | 2703.356 | 2703.323 | 12.21 | [WTPEVKHFCPNVPIILVGNKK] | [104-124] | T105-C112 |

**Table S16:** List of identified side products of cross-linking reaction of RHOA with **TR2** without ascorbate activation.

| Togni cross-linking reagent | Type of product | Exp. Mass | Theor. Mass | Deviation in ppm | Sequence | Modified peptides | Modified residues |
|---|---|---|---|---|---|---|---|
| TR2 | Type 2 | 2806.377 | 2806.364 | 4.55 | [GPLGSMAAIR]-[HFCPNVPIILVGNK] | [1-10]-[110-123] | G1-C112 |
| TR2 | Type 2 | 2234.933 | 2234.932 | 0.32 | [IGAFGYMECSAK]-[TKDGVR] | [156-167]-[168-173] | C164-K169 |
| TR2 | Type 1 | 2703.356 | 2703.323 | 12.21 | [WTPEVKHFCPNVPIILVGNKK] | [104-124] | T105-C112 |

**Table S17:** List of identified Type 0 side products of cross-linking reaction of RHOA with **TR2** using FFAP.

| Togni cross-linking reagent | Type of product | Exp. Mass | Theor. Mass | Deviation in ppm | Sequence | Modified peptides | Modified residues |
|---|---|---|---|---|---|---|---|
| TR2 | Type S0a | 1333.563 | 1333.557 | -4.89 | [LVIVGDGACGK] | [13-23] | C21 |
| TR2 | Type S0a | 2310.941 | 2310.926 | -6.35 | [QVELALWDTAGQEDYDR] | [57-73] | W63 |
| TR2 | Type S0a | 1061.407 | 1061.405 | -1.95 | [WTPEVK] | [104-109] | W104 |
| TR2 | Type S0a | 1852.853 | 1852.852 | -0.49 | [HFCPNVPIILVGNK] | [110-123] | C112 |
| TR2 | Type S0a | 1594.576 | 1594.566 | -6.15 | [IGAFGYMECSAK] +1 oxidation | [156-167] | C164 |
| TR2 | Type S0a | 1284.473 | 1284.468 | -4.42 | [EVFEMATR] | [174-181] | F176 |
| TR2 | Type S0a | 1021.415 | 1021.413 | -1.70 | [SGCLVLK] | [193-199] | C195 |
| TR2 | Type S0c | 1448.555 | 1448.547 | -5.53 | [GPLGSMAAIR] + 1 oxidation | [1-10] | G1 |
| TR2 | Type S0c | 1135.410 | 1135.401 | -7.79 | [TKDGVR] | [168-173] | K169 |

**Table S18:** List of identified Type 0 side products of cross-linking reaction of RHOA with **TR2** without ascorbic acid activation.

| Togni cross-linking reagent | Type of product | Exp. Mass | Theor. Mass | Deviation in ppm | Sequence | Modified peptides | Modified residues |
|---|---|---|---|---|---|---|---|
| TR2 | Type S0a | 1461.654 | 1461.652 | -1.78 | [KLVIVGDGACGK] | [12-23] | C21 |
| TR2 | Type S0a | 2310.928 | 2310.926 | -0.63 | [QVELALWDTAGQEDYDR] | [57-73] | W63 |
| TR2 | Type S0a | 1852.849 | 1852.853 | 2.53 | [HFCPNVPIILVGNK] | [110-123] | C112 |
| TR2 | Type S0a | 1594.578 | 1594.566 | -7.08 | [IGAFGYMECSAK] +1 oxidation | [156-167] | C164 |
| TR2 | Type S0a | 1284.473 | 1284.468 | -4.48 | [EVFEMATR] | [174-181] | F176 |
| TR2 | Type S0a | 1021.415 | 1021.413 | -1.62 | [SGCLVLK] | [193-199] | C195 |
| TR2 | Type S0b | 1851.868 | 1851.869 | 0.27 | [HFCPNVPIILVGNK] | [110-123] | C112 |
| TR2 | Type S0b | 1020.434 | 1020.429 | -4.52 | [SGCLVLK] | [193-199] | C195 |
